# Health systems response to climate change adaptation: a scoping review of global evidence

**DOI:** 10.1186/s12889-024-19459-w

**Published:** 2024-07-29

**Authors:** Edward Wilson Ansah, Mustapha Amoadu, Paul Obeng, Jacob Owusu Sarfo

**Affiliations:** Department of Health, Physical Education and Recreation, University of Cape Coast, Cape Coast, Ghana

**Keywords:** Health systems resilience, Adaptation, Barriers, Climate change, Review

## Abstract

**Background:**

The health system plays a critical role in safeguarding the well-being of communities in the face of health risks associated with climate change. This review maps evidence on health systems' adaptation to climate risk and barriers to effective adaptation.

**Methods:**

This review followed the recommendations by Arksey and O’Malley for conducting scoping review. Search for records was conducted in PubMed, Central, Web of Science, JSTOR, Google, and Google Scholar. Only peer-reviewed papers published in English language were included in this review. All the 63 included studies were critically appraise d.

**Results:**

We found that efforts are being made to create resilient health systems by incorporating climate change into health policies. Investments are being made in innovative technologies, climate-resilient health infrastructure, enhancing healthcare delivery, developing the capacity of climate specialists and agencies to provide high-quality evidence for resilient health systems. We also found that several obstacles prevent health system adaptation to climate risk, including poor policy implementation and evaluation. The obstacles are further exacerbated by financial constraints, including poverty, a lack of political commitment, inadequate data, and deficient healthcare systems, especially in developing countries. There is also a lack of integration of climate change into mental health actions and the health and safety of healthcare workers.

**Conclusion:**

Efforts to develop resilient health systems against climate risks are underway, but persistent obstacles, including inadequate policy implementation, resource limitations, and a lack of integration of climate change into critical health domains, hinder comprehensive adaptation measures, particularly in developing nations.

**Supplementary Information:**

The online version contains supplementary material available at 10.1186/s12889-024-19459-w.

## Introduction

Climate change is one of the most critical global defining phenomena of the 21^st^ century [1, 2]. According to World Health Organisation (WHO) climate change is the greatest threat to global health of our century [3]. Thus, anthropogenic climate change poses significant threats to various aspects of human life, including health, food security, sanitation, livelihoods, essential service delivery such as health service delivery, which impacts biodiversity loss and environmental degradation which also impacts human health through different pathways [3]. It is important to note that climate change is not just an environmental crisis, it is also a health crisis that demands global actions, effective partnerships and innovative solutions [4]. The health crisis from climate change has become increasingly evident in recent decades [2, 5]. Increased heat-related illnesses, altered disease patterns, compromised food and water security, and mental distress are some effects of the changing climate [4, 6, 7]. Higher risk populations, including the older population, pregnant women, migrants, newborns, children, those living in low-income communities, indigenous people and people experiencing homelessness, are disproportionately affected by the climate crisis [4].

There is an urgent need to protect these at-risk population via public health adaptation to climate change, and the health system plays a key role [8]. Thus, effective mitigation and adaptation strategies are needed. Climate change mitigation refers to efforts aimed at reducing or preventing the emission of greenhouse gases into the atmosphere or enhancing their removal from it. This includes actions such as transitioning to renewable energy sources, improving energy efficiency, and implementing policies to reduce deforestation [1–3]. On the other hand, climate change adaptation involves actions taken to minimise the negative impacts of climate change and to build resilience to its effects. This can include measures such as constructing flood defenses, developing drought-resistant crops, and implementing early warning systems for extreme weather events [1–3].

The health system plays a crucial role in safeguarding the well-being and resilience of communities in the face of health risks associated with climate change [9]. Health system comprises a wide range of health institutions, policies, and resources that collectively deliver healthcare services [10]. It includes not only healthcare providers and facilities, but also extends to environmental and sanitation services, health promotion and education initiatives, public health function and services, community health, healthcare supply chains, long-term care, health financing mechanisms, and other supporting elements. Additionally, the health system involves various stakeholders such as governments, regulatory bodies, non-governmental organisations (NGOs), community health workers, and healthcare professionals, all playing critical roles in ensuring effective healthcare delivery and improving population health outcomes. By addressing the broader determinants of health and incorporating a holistic approach, a comprehensive health system strives to provide not just accessible, equitable, and quality care to individuals and communities, but quality preventative and public health services [11].

The health system, positioned at the forefront of safeguarding and promoting public health, is an indispensable component of broader climate action strategies [11]. The health system plays a critical role in all climate actions due to its interconnectedness with various climate-related issues such as public health risks, disease prevention, emergency response, and healthcare delivery to at-risk populations [3]. Hence, building a resilient health system globally may play a pivotal role in safeguarding and promoting public health. The WHO [3] Health System Resilience Framework defines health system resilience as "the capacity of health actors, institutions, and populations to prepare for and effectively respond to crises; maintain core functions when a crisis hits; and, informed by lessons learned during the crisis, reorganise if conditions require it” [3]. The WHO Health System Resilience Framework [3] and the new Operational Framework for Climate Resilient and Low Carbon Health Systems [2] encompasses several key components vital to understanding and fortifying health system resilience to the climate crisis. These components typically include sustainable financing, governance and leadership, health workforce and service delivery, health information systems, medical products and technologies, community engagement and reduction in carbon emission. Understanding these elements is crucial for evaluating a health system's resilience, inducing the ability to adapt and respond effectively to various challenges, including those posed by climate change.

Resilient health systems can absorb and adapt to the challenges posed by climate change while ensuring the provision of essential health services, protecting high risk or vulnerable populations, and promoting sustainable well-being [2, 3]. By examining how health systems respond to the challenges posed by climate change, this review aligns with the WHO framework's emphasis on a system's capacity to prepare for, respond to, and recover from climate crises [3]. Highlighting the impacts of climate change on public health, such as heat-related illnesses, altered disease patterns, and compromised food security, underscores the urgent need for health systems to enhance their resilience [3]. Understanding how health systems worldwide adapt to these challenges becomes pivotal in formulating evidence-based strategies that align with the core principles of health system resilience outlined in the new operational framework by WHO [2]. With this new WHO operational framework, we have a dual responsibility to build health systems that can withstand climate-related shocks, while at the same time reducing their carbon footprint [2].

Mapping climate adaptation strategies by health systems globally may be an essential effort in providing evidence important to inform evidence-based decision-making, identify best practices, and address the gaps and challenges in current responses to climate change. Hence, this scoping review seeks to identify health system response, and barriers or challenges of the current responses to climate change within health systems. This review aims to contribute to the development of robust and context-specific policies that enhance building climate resilient health systems and the communities they serve. In developing countries where health system adaptation faces significant challenges, this review seeks to provide valuable insights for developing efficient, and cost-effective solutions. Thus, solutions that align with the WHO's resilience framework, ensuring the continuity of essential health services amidst climate-related crisis.

## Methods

This scoping review followed the guidelines of Arksey and O'Malley [12]. The guidelines include identifying research questions or objectives, searching for relevant studies, selecting studies, extracting data, summary of data and synthesis of results, and consultation. This review adheres to PRISMA-ScR guidelines of conducting and reporting scoping reviews. Arksey and O'Malley's framework provides a systematic process for scoping reviews, ensuring comprehensive coverage of research areas and structured methodology. Moreover. PRISMA-ScR guidelines offer standardised reporting criteria, enhancing transparency and replicability of scoping review findings. Two research questions guided this review: (1) what are the health system adaptation strategies to climate change? and (2) what are the barriers to health system responses to effective adaptation to climate change? The search for relevant studies was conducted in four databases (PubMed, Central, Web of Science, and JSTOR). The search was initially conducted in PubMed with Medical Subject Headings (MeSH) terms (see Table 1). These MeSH terms were then modified to suit the search in other databases (Central, Web of Science and JSTOR). An additional search was conducted in Intergovernmental Panel on Climate Change (IPCC) library, WHO library, Google, and Google Scholar. The last search in all databases was conducted June 30, 2023.

**Table 1:** Planned search strategy in PubMed

Search strategy to identify publications focused heat-related illness	
It identifies health system ***#1***	(Health system*[MeSH Term] OR Healthcare system* OR Medical system OR Health services* OR Healthcare delivery system* OR Health infrastructure* OR Health sector* OR Healthcare network* OR Medical care system* OR Health organization* OR Public health system* OR Health facilities* OR Healthcare institutions* OR Health services sector* OR Health management system* OR Health service delivery* OR Public health* OR primary healthcare* OR Hospital*community engagement* OR Sanitation management* OR Waste Management* OR Water management* OR Health information system* OR Health financing* OR Health workforce* Or Health policies*)
It identifies climate hazard ***#2***	(Climate change*[MeSH Term] OR air temperature* OR climate variability* OR global warming* OR heat* OR hot temperature* OR heat wave* OR Air pollution* OR Disasters* OR Extreme weather events* OR Environmental exposure* OR Rising temperature* OR Climate crisis* OR Climate disruption* OR Environmental change* OR Climate fluctuations* OR Climate shocks* OR Climate shifts* OR Greenhouse effect*)
It identifies the Adaptation ***#3***	(Adaptation*[MeSH Term] OR Mitigation* OR Resilience* OR Response* OR Solution* OR adjustment* OR Evolution* Preparedness* OR Risk management* Actions* OR Measures* OR Risk reduction* OR Protection* OR Planning* OR Strategies* OR Measures* OR Coping* OR Reduction* OR Initiatives* OR Policies*)
Overall search strategy	***#***1 AND ***#***2 AND ***#***3 NOT animal* (Filters activated: English, from 2000/01/01 to 2023/06/06)

Relevant records were then transferred to the Mendeley software to remove duplicate papers. Titles and abstracts of papers were screened for relevance. This was done by 20 trained graduate students and supervised by the authors. The 20 trained graduate assistants were put into two groups, each made up of 10. These two groups screened the titles and abstracts independently using the eligibility criteria. The students were supervised by M.A and JOS. Weekly meetings were used to resolve inconsistencies and disagreements by the help of EWA. Reference lists of full-text records were further checked for other relevant papers. Full-text papers were then screened based on the eligibility criteria, presented in Table 2. Full-text records were screen independently by MA and PO and reviewed by JOS and EWA. Data were extracted independently by MA and PO and reviewed by JOS and EWA. This was done to ensure that extracted papers were reliable and accurate. Moreover, authors (JOS and EWA) resolved inconsistencies between extractors during regular meetings. Furthermore, an independent researcher, review and subject expert reviewed the extracted data for accuracy. We extracted the data based on authors, year of publication, the purpose of study, study design, health system response to climate change, barriers to adaptation and critically appraised the studies.

**Table 2:** Eligibility criteria used for screening

Items	Criteria
Databases	PubMed, Central, Web of Science, JSTOR, IPCC library, Google and Google Scholar
Time filter	The year 2000 or later
Spatial filter	Global
Language filter	English language
Inclusion criteria	The paper should be: 1. a peer-review paper, 2. conducted using primary data, review or policy analysis 3. a study conducted on health system response or adaptation to climate change, 4. published in English language
Exclusion	The paper should be: 1. a grey literature, an abstract, conference paper, letter to editor and preprint 2. a paper conducted outside the scope of health system adaptation to climate change 3. published in any language other than English

Briggs’s Critical Appraisal Tools, developed and updated by Joanna Briggs Institute in 2020 were used to appraise reviewed studies. These tools were very recently employed in a similar study [13]. The aim was to appraise all selected and reviewed studies. This tool comprises checklists for evaluating the quality of qualitative studies, cross-sectional studies, mixed-method designs and reviews. Mixed Method Appraisal Tool (MMAT) version 2018 was used to appraise all included mixed-method studies [14]. Appraisals were conducted by MA, PO and JOS, supervised by EWA. Extracted data was analysed using thematic content analysis, summarised and qualitatively synthesised as recommended Arksey and O’Malley [12]. The main purpose was to map existing evidence and hence qualitative synthesis was appropriate. This scoping review was registered with Open Science Framework [10.17605/osf.io/kt7bq].

### Search results

**Fig. 1:**
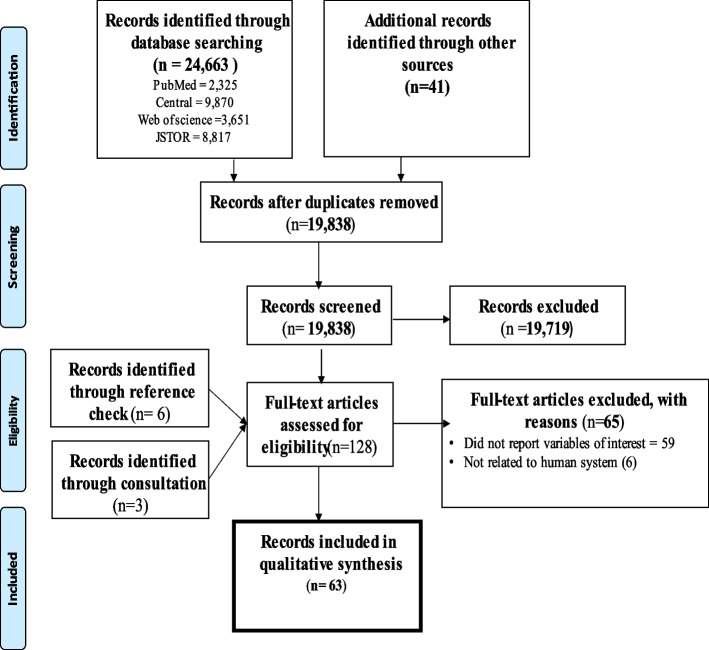
PRISMA flow diagram of search results and screening process

Search conducted in PubMed, PubMed Central, Web of Science and JSTOR produced 24,663 records. Additional 41 records were retrieved from other databases. These records were saved in the Mendeley software, and 4866 duplicate records were removed. Titles and abstracts of remaining records were screened for eligible full-text papers. This resulted in the removal of 19,719 records that were considered not eligible. There were 119 remaining eligible full-text papers for further screening. Reference lists of these records were further checked for additional records and 6 full-text papers were identified. Through consultation with the digital library department at Sam Jonah Library, additional 3 full-text records were retrieved. Hence, 128 full-text papers were assessed. Finally, 63 full-text papers were included in this review. Details of search records and screening process are presented in Fig. 1.

### Characteristics of reviewed studies

**Fig. 2:**
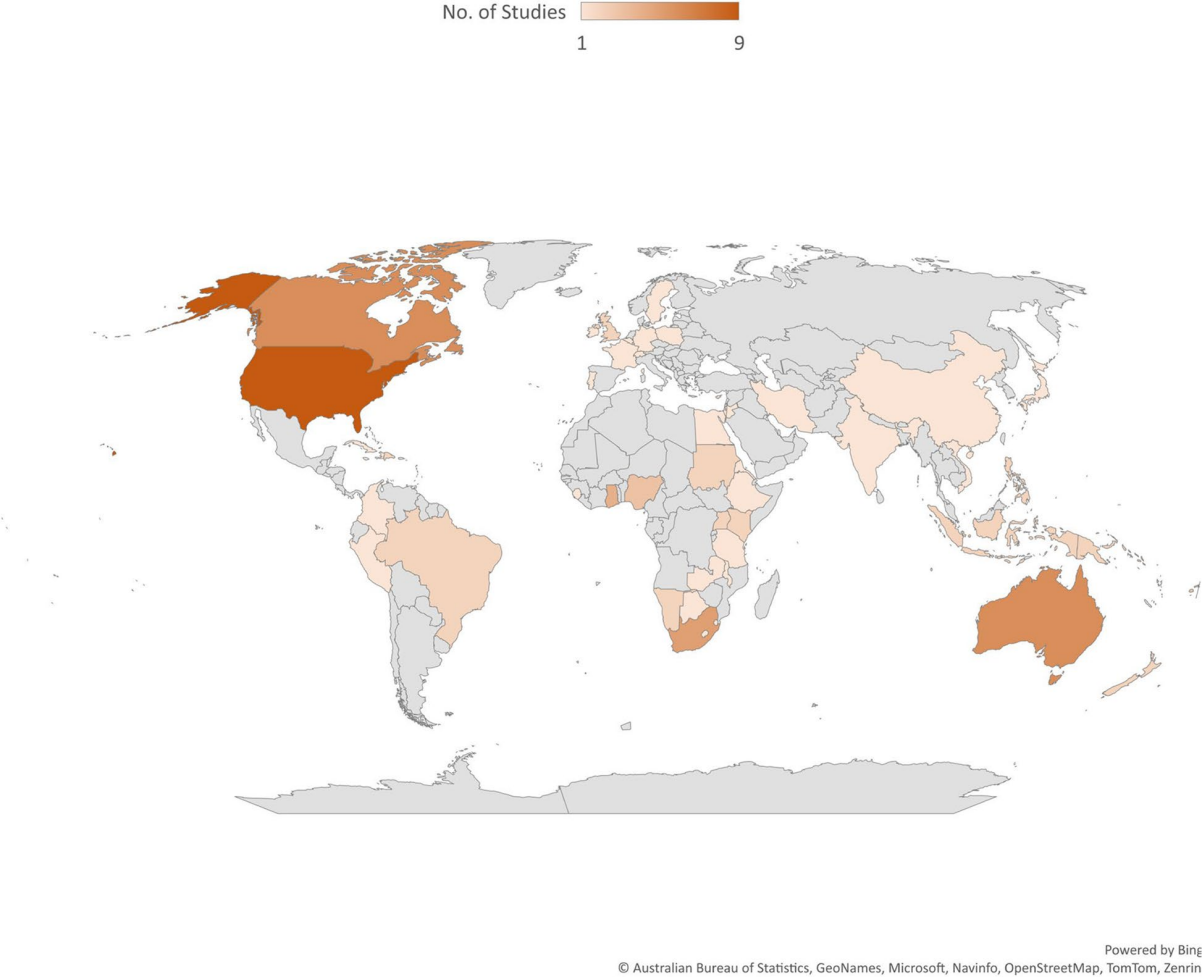
Countries where reviewed studies were conducted

Most (46) of the reviewed studies were conducted on policy documents and studies that explored health systems' response to climate change in various countries and regions. The reviewed studies included 46 reviews, eight mix-method design studies, seven qualitative design studies and two cross-sectional design studies. Furthermore, we retrieved studies conducted in 85 countries globally, with the United States of America recording the highest number of studies (9). Figure 2 shows the countries and continents where these studies were conducted. The characteristics of the studies reviewed are presented in Supplementary File (Table S1).

## Results

Findings are presented in themes based on the research questions.

### Health system response to climate change

**Fig. 3:**
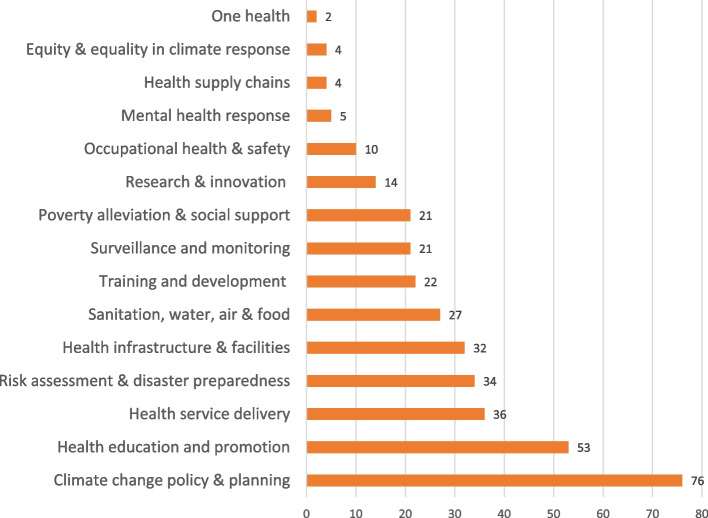
Weights assigned to themes generated from the included studies

Forty-six (46) included studies with varied designs (cross-sectional surveys, qualitative, mixed-method and document review) presented findings on health system response to climate change. Through thematic content analysis, health system responses to climate change were grouped into 15 themes. Frequency counts were assigned to each theme based on the specific adaptation actions and the number of studies that explored such actions. For instance, there are 23 specific climate adaptation actions under the theme “climate change policy and planning”. Studies that explored each action are then added to give counts to the theme. For example, a study that explored five specific actions under a theme were counted as five. Figure 3 presents the themes generated from the reviewed studies and the assigned counts. Table 3 presents the themes and specific climate actions by health systems highlighted in reviewed studies.

**Table 3:** Themes generated and specific climate actions by health systems

Main theme	Specific responses	Authors
Health promotion	Create health promotion environment	[9, 15–21]
	Public education for individuals, families and communities on health impact of climate change including mental health issues	[8, 15, 17, 19, 20, 22–44]
	Promoting climate informed health programmes	[6, 18]
	Health education for health professionals	[27, 29, 45–47]
	Facilitates communities access to health programmes	[18, 26, 29, 46]
	Health promotion to reduce the burden of chronic disease	[24]
	Strengthen the case for investment in health promotion	[48]
	Communicating co-benefits and actions to protect health	[10, 18, 49]
	Mobilize community partnership and action	[34]
Disaster preparedness/ Risk assessment	Risk assessment, emergency planning and preparedness	[9, 19, 21, 29, 31, 36, 40, 41, 50–53]
	Coordinate disaster response emergency services	[38]
	Assessment of health impact	[15, 54]
	Vulnerability assessment including the health system	[18, 28, 33, 48, 50, 53–55]
	Emergency risk communication	[9, 29, 50, 53, 55]
	Providing emergency management stakeholders with reliable information	[48]
	Health messaging to critical periods	[9]
	Rapid disease specific emergency response	[29]
	Integrated assessment model	[54]
	Adequate funding for disaster preparedness	[9, 56]
Surveillance & Monitoring	Routine epidemiologic surveillance	[9, 15, 18–20, 22, 24, 25, 28, 29, 34, 36, 37, 39, 41, 52, 55, 57]
	Syndromic surveillance	[54]
	Enhancement of surveillance programmes to include climate sensitive disease and their risk sources	[18, 32, 45, 56, 58]
	Early warning system	[6, 8–10, 13, 16–18, 21, 22, 26, 29, 31–33, 36, 38, 40, 41, 45, 59–61]
	Remote sensing	[54]
	Implement new and effective early warning systems technologies	[29, 33, 55]
	Specific disease and vector programmes	[58, 62, 63]
	Eradication of Aedes japonicus mosquito	[27]
	Mosquito control programmes	[9, 55]
	Improving vaccine/vaccination programmes	[25, 28, 36, 40]
	Immunization programmes for at risk communities and children	[64]
	Screening of border crossers	[8]
	Point of care diagnostics for disease outbreaks	[30]
	Mobile devices for surveillance	[30]
	Developing robust diagnosis for food and water borne diseases	[27, 40]
	Make available testing and diagnosis in vulnerable communities	[44]
Mental health response	Monitoring psychosocial resources and skills in communities	[65]
	Mapping inter-disciplinary relations for psychosocial response	[65]
	Providing resources and information for mental health adaptation	[44, 65]
	Increase awareness of mental health effect of climate change among health workers	[44]
Social support systems	Temporary shelter for displaced people	[19]
	Timely relocation of displaced people and migrants	[29, 57]
	Maintain social structure for forced migration	[29]
	Improving housing systems for lower-income families	[25, 29, 41]
	Evacuation of vulnerable population	[19]
	Neighbourhood support schemes	[36, 39]
	Facilitating organised relocation	[8]
	Enhancing legal and effective migration	[8, 32]
	Provision of alternative employment opportunities	[8]
	Provide migrants with adequate nutrition, shelter etc	[8]
	Relief plans for vulnerable people	[10, 38]
	Support social network	[10]
	Poverty alleviation programmes	[18, 41]
	Prevent heat waves in vulnerable people	[61]
Health service delivery	Outreach targeting vulnerable population and home care	[19, 39, 44, 58, 63]
	Facilitate access to healthcare	[22, 49, 58, 64]
	Early treatment of infections	[58]
	Strengthen health system in climate hotspots	[8, 53]
	Telemedicine/eHealth	[30]
	Prepare health services for emergencies	[27, 64, 66]
	Strategic allocation of health resources	[66]
	Provide culturally-appropriate services	[34, 41, 49, 64]
	Patient-centered care	[17, 34, 49]
	Improve management of climate sensitive diseases	[23]
	Develop community-based models for the management of children with acute malnutrition	[52]
	Expanding emergency services to include under-five children	[18]
	Improve access to pharmaceuticals for increased health risk	[18]
	Improve capacity and quality of care	[18, 33, 34, 44, 49]
	Community-oriented approach to healthcare	[34, 47]
Health infrastructure/facilities	Improve health infrastructure and facilities	[8–10, 14, 18, 21, 26, 28, 29, 38, 43, 45, 53, 54, 60, 61,67
	Improve ventilation	[10, 17, 20, 30]
	Reviewing cooling capacity in all health facilities	[31]
	Ensuring thermal comfort	[17]
	Making health infrastructure robust to heat stress	[59]
	Modernisation of laboratories for early diagnosis	[9, 20]
	Build social infrastructure to support care system	[21]
	Support-of-the grid solutions	[31, 33, 47]
	Back-up generator for emergency solutions	[44, 47]
Health supply chains	Rapid assessment to identify needed resources	[19]
	Stockpile medical supplies and pharmaceuticals	[20, 27]
	Improve health supply chains	[29]
Training and development	Training and capacity building of health workforce	[6, 8, 9, 18, 20, 22, 23, 28, 33, 36, 45, 47, 50, 55, 57]
	Prepare frontline health workers to manage heat stress	[44]
	Strengthen network and capacity of expertise and centres	[18, 27, 28, 53, 68]
	Periodic courses to upskill personnel in diagnosis and treatment	[20]
Research and innovation	Improve scientific research/evidence	[22, 23, 25, 33, 34, 48]
	Sponsor research for vaccines	[40]
	Funding for quality research	[16, 20]
	Translate research into practice/ knowledge dissemination	[18, 69]
	Policy oriented research	[64]
	Promote research	[28]
	Increasing tree canopy	[55]
Sanitation, water, air and food	Assessment of sanitation, water, air and food	[19, 36, 40]
	Nutritional supplement/programmes	[18, 36]
	Maintaining and improving water systems and sources	[9, 24, 28, 30, 52, 61, 63]
	Improve waste management	[9, 25, 30]
	Invest in clean water technologies	[25]
	Improving sanitation/hygiene infrastructure and activities	[10, 28, 52, 56, 61, 63]
	Access to clean and safe water and food	[41, 56]
	Proper food handing certification and enforcement	[9, 24]
	Community clean-up campaigns	[9]
One health	Adopting one health approach	[8, 66]
Address Equity/equality	Equitable resources distribution	[8]
	Prioritising equity in health adaptation planning	[10]
	Increase work on the shared causes of climate change and inequities	[26]
	Ensure equitable access to health services	[44]
Climate policy/planning	Refine and develop regulatory framework for climate change	[8, 22–24, 45, 46]
	Long term planning	[22, 66]
	Collaboration with non-health sector/inter sector	[9, 10, 22, 23, 26, 27, 32, 46–48, 50, 56, 57, 59, 61, 64, 66, 67]
	Cross-sector coordination	[6, 22]
	Policy evaluation	[6, 15, 22, 28, 49, 50, 69]
	Policy on conserving energy in health facilities	[30]
	Renewable energy sources	[30]
	Reducing GHG emissions from anesthetic gas	[30]
	Cultivation of climbing plants	[39]
	Integrating hospitals into urban planning	[16, 17]
	Integrating health adaptation into national health planning	[9, 15, 16, 48, 55, 56, 59]
	Mainstreaming climate change in health policies	[25]
	Heat risk adaptation guidelines	[27]
	Integrating climate risk monitoring into health programmes	[6, 15]
	Sea defense project	[28, 29]
	Enforcing regulations on climate policies	[9, 28, 34]
	Develop national electronic base for climate-change issues	[9, 31, 46, 55]
	Health system business continuity planning	[70]
	Energy efficiency initiatives in health facilities	[70]
	Standardised health impact projection reporting	[54]
	Improve universal healthcare access	[18, 49]
	Adequate funding and resources for climate adaptation	[18, 26, 33, 46]
	Improve transportation to increase access to healthcare	[60]
	Integrate climate change education in schools and graduate studies	[16, 25, 26, 45]
Occupational health and safety	Improving staff number	[17]
	Work place ergonomics	[17]
	Climate change adapted work processes	[17]
	Investigate OHS implication of climate change	[27]
	Adequate protection of workers exposed to heat stress	[20, 31, 60]
	Incorporate climate change into OHS assessment	[31]
	Prepare and debrief staff on extreme events	[31]
	Insurance policies should cover extreme events for health workers	[31]

#### Health policy and planning

There are efforts being made especially in developed countries and some developing countries such as Vietnam [23], and some countries in SSA such as Ghana, Nigeria, Ethiopia, Namibia, Kenya and South Africa [45] to refine and establish regulatory frameworks for climate change actions in health policy [8, 22–24, 45, 46]. Furthermore, efforts are been made to mainstream climate considerations into all healthcare policies [25] and planning [9, 15, 16, 48, 55, 56, 59] at national and local levels. The importance of integrating hospitals into urban planning [16, 17] and incorporating climate change education into school curricula and graduate studies [16, 25, 26, 45] have also been highlighted. Studies have also emphasised the importance of climate change mitigating measures taken by the health systems, including reducing greenhouse gas (GHG) emissions from anaesthetic gas usage, promoting energy conservation in health facilities [30] and developing policies to improve transportation systems [60]. Moreover, evidence suggest that health systems are implementing essential policies aimed at heat risk adaptation [27], undertaking sea defence projects to protect coastal population and health infrastructures situated along the coastal areas [28, 29], and developing national electronic databases for climate change actions [9, 31, 46, 55] and ensuring universal healthcare access [18, 49] in responses to climate crisis. Policies aimed at facilitating effective climate change mitigation and adaptation within health systems rely on several key elements, including long-term planning [22, 66], collaborative efforts with non-health sectors [9, 10, 27, 28, 33, 35, 38, 39, 43, 47, 51, 52, 55, 58, 60, 61, 65, 69], sufficient funding and resources [18, 26, 33, 46], regular policy evaluation [6, 15, 22, 28, 49, 50, 69] and enforcement of policy regulations [9, 28, 34].

#### Health promotion

Health systems are making a strong case for investment in health promotion [48], by creating health promoting communities that fosters knowledge sharing on climate change and support health systems in several ways, including empowering local response and support at-risk populations [9, 15–21]. Health systems are focused on educating communities [8, 15, 17, 19, 20, 22–44] and health professionals [27, 29, 45–47] on the impact of climate change on human health and health systems. Promoting climate-informed health programmes [6, 18], facilitating communities' access to health programmes [18, 26, 29, 46] and communicating co-benefits and actions to protect the health of individuals and communities [10, 18, 49] are among important health promotion interventions adopted by health systems. Co-benefits encompasses additional health benefits derived from implementing climate-informed health programmes [18, 49].

#### Risk assessment, disaster preparedness and response

Health system response through risk assessment, emergency planning and preparedness were reported in the literature [9, 19, 21, 29, 31, 36, 40, 41, 50–53]. For instance, vulnerability assessments for health systems [18, 28, 33, 48, 50, 53–55], assessment of health impact [15, 54] and the use of an integrated assessment model [54] are adaptation strategies by health systems reported in included studies. Moreover, emergency risk communication, especially for high risk population and communities [9, 29, 50, 53, 55], including health messaging during extreme events [9] and rapid disease-specific emergency response are also essential health system adaptation strategies [29]. However, disaster response actions are more useful when they are well coordinated [38], planned based on reliable information [48] and adequately financed [9, 56].

#### Surveillance and monitoring of climate-sensitive diseases

Routine epidemiologic surveillance [9, 15, 18–20, 22, 24, 25, 28, 29, 34, 36, 37, 39, 41, 52, 55, 57], which enhances existing surveillance programmes to include climate-sensitive diseases [18, 32, 45, 56, 58], and the implementation of early warning systems [6, 8–10, 13, 16–18, 21, 22, 26, 29, 31–33, 36, 38, 40, 41, 45, 59–61] including innovative technologies like remote sensing [54] have been identified as valuable approaches. Many developed nations also emphasise disease-centric approaches, technological advancements, and are integrating climate change events into public health policies and planning [19, 20, 39]. Evidence emphasises particular disease and vector programmes [58, 62, 63], such as vaccination [25, 28, 36, 40], mosquito control [9, 55], and screening of pathogens at border or entry points [8], as effective actions within health systems which address climate change vulnerability. Immunisation programmes for at-risk communities and children, especially under-5 children, have been reported [64]. The importance of targeting at-risk communities and high risk populations through initiatives such as enhanced testing and diagnosis of climate-sensitive diseases [44], the utilisation of mobile devices for surveillance purposes [30], and the implementation of robust diagnostic methods for food and waterborne diseases [27, 40] have also been reported.

Furthermore, both developed and developing countries recognise the urgency of surveillance, research, and monitoring efforts in tackling climate-related health challenges. Developed nations are leveraging on advanced technology and resources for these purposes [39, 68], the developing countries are emphasisng capacity building and resource optimisation for effective surveillance and monitoring [25, 33, 64]. While these approaches differ based on resources and priorities, the common goal remains the adaptation and resilience of healthcare systems to address the impacts of climate change on public health.

#### Health service delivery and mental health

Improving health service capacity to deliver quality care [18, 33, 34, 44, 49], strengthening healthcare delivery in climate hotspots [8, 53] and preparing health services for emergencies [27, 64, 66] are effective ways to respond to the changing climate. Facilitating healthcare access [22, 49, 58, 64],including outreach that target at risk populations [19, 39, 44, 58, 63], telemedicine [30], and strategic allocation of health resources [66] are some essential climate change adaptation actions. Providing culturally appropriate [34, 41, 49, 64], community-oriented [34, 47] and patient-centred care [17, 34, 49] are effective means the health system deals with climate crisis. Also, developing community-based models for the management of children with acute malnutrition [52], improvement in the management of climate-sensitive diseases [23] and early treatment of infections [58] have been reported by included studies. In addition, providing resources and information for mental health adaptation [65], including monitoring of psychosocial resources and skills [44, 65] and creating awareness about the impact of climate on mental health, especially in high risk communities and healthcare workers [67] are essential to reduce the effects of changing climate on health systems.

#### Health infrastructure and supply chains

Across developed countries like the UK, USA, Australia, Poland, Canada, Germany, Japan, etc. notable similarities in health system responses to climate change emerged. These nations prioritise sophisticated infrastructure development and improvements, such as implementing early warning systems [15, 17, 21, 24, 27, 29, 55, 60, 70], developing comprehensive healthcare facility plans [68], and integrating the climate crisis into various healthcare programmes [17, 24]. Conversely, in developing nations like India, countries in SSA, and Southeast Asian, including Indonesia and Vietnam, the focus revolves around community-oriented approaches [19, 23]. These countries prioritize public education, access to clean water, sanitation, and collaboration across sectors [25, 33, 45, 57, 58]. Their adaptation strategies often revolve around building resilience in public health infrastructure, addressing poverty, and enhancing traditional healthcare systems [25, 33, 51, 58, 64]. Additionally, they highlight the importance of localised solutions and training healthcare workers to adapt to climate change impacts.

Health system response to climate change includes improving health infrastructure and facilities such as upgrading emergency response units, ensuring the resilience of medical equipment to extreme weather conditions, and establishing backup power sources for uninterrupted healthcare delivery during climate-related events [8, 10, 14, 45, 26, 28, 29, 60, 18, 67, 61, 21, 38, 43, 53, 54]. Others include modernisation of laboratories for early diagnosis [9, 20], building social infrastructures such as community health centers, support groups for at-risk populations, mental health hotlines, neighborhood networks that promote health education, and community-based organisations that support care systems [21] and supporting off-the-grid solutions for hospitals [31, 33, 47]. Ensuring thermal comfort [17] through improved ventilation [10, 17, 20, 30] is vital in healthcare facilities in rising temperatures. Health systems are prioritising improvement in health supply chains [29] that are less pollutant through rapid assessment to identify needed supplies [19] and stockpile medical supplies and pharmaceuticals [20, 27].

#### Social support systems and equity

Developing poverty alleviation programmes [18, 41], provision of alternative employment opportunities [8] and improving housing systems for lower-income families [25, 29, 41] are relevant social interventions that aid health system adaptations. Moreover, evacuation of high risk populations [19], including timely relocation of displaced people and migrants [29, 57] and provision of temporary shelter for displaced people [19] are actions needed for the protection of vulnerable people for improved health outcomes. Extreme weather events necessitate neighbourhood support schemes [36, 39], improved social networks [10] as well as relief programmes for the population at risk of climate change events [10, 38], including people living in hard-to-reach areas. Efforts are being made to preserve social structures for populations facing forced migration [29], to enhance legal and effective migration [8, 32], facilitate organised relocation [8] and provide migrants with adequate nutrition and access to healthcare [8]. Health system adaptation to climate change may help reduce issues of inequity and ensures equitable access to healthcare [44] through equitable distribution of health resources [8].

#### Research, training and development

Health system adaptation to climate crisis includes improving scientific research [22, 23, 25, 33, 34, 48], provision of funding for quality research [16, 20] and vaccines [40] and the translation of research into practice [18, 69] for quality health services. Besides, policy-oriented research is essential in supporting effective health system adaptation to climate change [64]. Nations are training and building the capacity of their health workforce on climate change and its impact on health [6, 8, 9, 18, 20, 22, 23, 28, 33, 36, 45, 47, 50, 55, 57], preparing frontline health workers to manage heat stress [44] and strengthening network and capacity of experts and institutions [18, 27, 28, 53, 68].

#### Sanitation, water and food

Efforts are being directed towards improving sanitation through improving waste management [9, 25, 30] and hygiene infrastructure [10, 28, 52, 56, 61, 63], as well as promoting community clean-up campaigns [9]. Increasing access to clean food and water [41, 56] through maintaining and improving water systems and sources [9, 24, 28, 30, 52, 61, 63]. Others are investing in clean water technologies [25] and enforcing proper food handling regulations systems [9, 24].

#### One Health

One health refers to an interdisciplinary approach that recognises the interconnectedness of human health, animal health, and the environment [8]. Thus, one health emphasises the interdependencies between the health of humans, animals, and ecosystems and recognises that their well-being is closely intertwined. The importance of health system adaptation to climate change through one health has been emphasised [8, 66]. One Health emphasises the interconnectedness of human, animal, and environmental health, advocating for collaborative approaches to address the impacts of climate change on health systems [66].

#### Occupational health and safety (OHS)

The importance of incorporating climate change effects into OHS assessment [31] and increased research on OHS implications of climate change [27] are paramount to health system adaptation to climate change. Ensuring safety and well-being of healthcare workers involve implementing measures that protect workers from heat stress [20, 31, 60], optimising workplace ergonomics [17], enhancing staffing levels [17] increasing staff insurance policies [31], and adopting work processes that are adapted to the challenges posed by climate change [17].

Our findings encompass a broader spectrum of strategies implemented by health systems in response to climate change. While the WHO framework primarily focuses on specific categories like governance, leadership, health workforce, service delivery, information systems, essential medicines, financing, and research, our research delves deeper into additional areas such as social support systems, One Health strategies, and OHS. These expansions transcend the defined categories of the WHO framework, showcasing a more comprehensive understanding and implementation of health system responses to climate change across various interconnected domains.

### Barriers to health system response to climate change

**Fig. 4:**
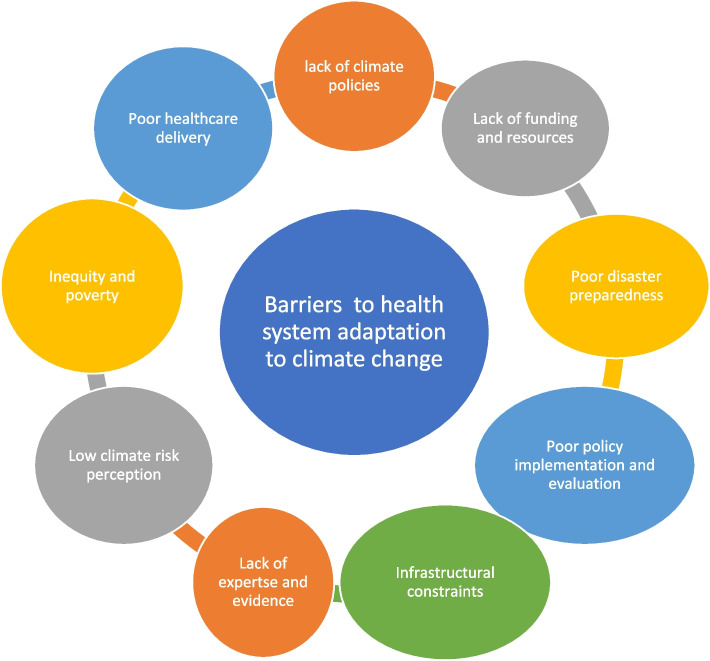
Barriers to health system adaptation to climate change

Twenty-nine included studies reported on barriers to effective adaptation to climate change by health systems. The thematic analysis of the included studies yielded nine distinct themes on the barriers to health system adaptation to climate crisis. These themes include inadequate climate policies and disaster preparedness, resources constraints, poor policy implementation and evaluation, low-risk perception, lack of expertise and evidence, inequity and problems in healthcare delivery. These themes are presented in Table 4 and Fig. 4.

**Table 4:** Barriers to health system response to climate change

Main theme	Barriers	Authors
Lack of climate policy	Lack of supportive policy environment	[19]
	Lack of prioritization of climate change adaptation actions	[71]
	Lack of incorporating climate and health into planning	[22]
	Limited plans and programmes to tackle health risk	[59]
	Adaptation initiatives do not target specific health risks	[59, 72]
	Lack of adaptation policies for mental health	[59]
	No policy paper in Tanzania is solely responsible for climate change	[51]
	Uncertainty in climate projections and best adaptation options	[9, 48, 54]
	Fragmented and policy contradictions	[72, 73]
	Unclear long-term planning	[64]
	National adaptation plan does not include health as a priority	[72]
	Few adaptation policies on OHS	[62]
	Climate change is not in the mainstream curricula of medical school	[62]
	A narrow framework	[26]
Poor disaster preparedness	Slow disaster preparedness in hospitals and clinics	[30]
	Lack of resources planning in disaster response	[59]
	Little efforts on preparedness for extreme weather events	[62]
	Not prepared for the burden of climate migration	[64]
	Lack of surveillance on vulnerabilities	[59]
Poor policy implementation and Evaluation	Ineffective coordination mechanisms	[6]
	Lack of monitoring and evaluation	[6, 72]
	Unsuccessful implementations of interventions	[6]
	Lack of strategic implementation	[51]
	Institution arrangements limits collaborative efforts	[54, 61, 73]
	Limited regional collaborations	[64]
Lack of funding and resources	Lack of financial resources	[9, 26, 35, 48, 54, 61, 71–73]
	Lack of long-term funding	[15]
	Lack of political will or support	[35, 62, 64]
	Difficulty mobilising resources	[6, 15]
	Lack of investment in health system	[64]
Infrastructural constraints	Lack of infrastructure for robust mobile communications	[30]
	Lack of alternative and renewable supply	[30]
	New technologies remain underdeveloped and expensive	[30, 54, 68, 73]
	Poor infrastructure	[59]
Lack of expertise and evidence	Perceived lack of expertise	[39, 64, 71]
	Lack of expertise in developing countries	[48]
	Lack of skilled workforce	[9, 30]
	Lack of education for skilled workforce	[30]
	Lack of locally relevant evidence on efficacy of interventions	[9, 15, 39, 69, 73]
	Little research on climate change and health impact	[51]
	Insufficient training of stakeholders on climate health risk	[9]
	Difficulty to access climate data	[48]
	Limited climate change and health models	[48]
	Lack of institutional capacity	[68]
	Lack of information on complexity nature of disease transmission	[58]
	Lack of information on the link between climate change and health	[33, 37]
	Overload of information	[6]
	Information deficit	[33, 68]
	Gaps in reporting	[6]
	Effective messaging is a problem in diverse communities	[15]
	Lack of guidelines for climate change health impact reporting	[69, 72]
	Lack of information on adaptation initiatives	[33]
	Lack of public health campaigns	[26]
Low risk perception	Low climate risk perception	[15, 26, 35, 73]
	Lack of knowledge of health risk from climate change	[9, 26, 74]
	Wrong perception about climate change health impact	[15, 54, 58]
	Lack of awareness about heat stress among healthcare workers	[59]
	Lack of urgency	[15]
Inequity and poverty	Social inequality	[58]
	Maldistribution of adaptive capacity	[54]
	Socio-political inequality	[68]
	Health inequalities	[74]
	Fragmented services for migrants	[64]
	Socio-economic challenges	[54, 73]
	Economic poverty	[68]
	Little consideration is given to marginalized people	[62, 72]
Problems in healthcare delivery	Lack of treatment protocols for illness related to extreme events	[59]
	Shortage of staff	[6, 59]
	Issues in integrating evidence-based practices into healthcare practices	[6]
	Strained health system	[62]

#### Inadequate climate policies and disaster preparedness

Lack of a supportive policy environment [19], inadequate prioritisation of climate change adaptation actions [71], and limited integration of climate and health issues into planning [22] were policy issues that make health system adaptation to the changing climate difficult. Furthermore, there were limited plans and programmes to address health risks associated with climate change [59], and existing adaptation initiatives often did not target these risks [59, 72]. For instance, a lack of adaptation policies for mental health [59] was reported. Evidence indicates that uncertainty surrounding climate projections and the best adaptation options [9, 48, 54], along with fragmented policies and contradictions [72, 73], present further challenges to health system adaptation. Moreover, the indication is that unclear long-term planning [64], and national adaptation plans do not prioritise health [72]. Few adaptation policies were found concerning OHS [62], but climate change was not integrated into the mainstream curricula of medical schools [62], indicating a narrow framework of climate policies [26] that may affect climate action implementation and evaluation. Furthermore, lack of preparedness for the burden of climate migration [64] and insufficient surveillance and assessment of vulnerabilities associated with climate change impacts [59] are challenging health system attempt to adapt effectively to the climate crisis.

#### Resources constraints

Insufficient financial resources [9, 26, 35, 48, 54, 61, 71–73] coupled with the challenge of securing long-term funding [15] hinder health system adaptation to climate change. Moreover, slow disaster preparedness in health facilities [30], lack of resource planning for disaster response [59], and limited efforts in preparing for extreme weather events were also reported [62]. Inadequate political will or support [35, 62, 64] further exacerbates this situation, which limits the allocation of resources and impedes the implementation of effective strategies. The difficulty in mobilising resources [6, 15] and the dearth of investments in the health system [64] amplify the constraints faced in responding to climate-related health risks. Additionally, the presence of poor infrastructure [59] and the introduction of underdeveloped and expensive new technologies [30, 54, 68, 73] pose significant barriers, that hinder the ability of health systems to effectively adapt to the challenges posed by climate change.

#### Poor policy implementation and evaluation

The effectiveness of health system adaptation programmes and policies is significantly hindered by inadequate coordination mechanisms among stakeholders [6], resulting in a lack of synchronised efforts to address climate-related health challenges. This lack of harmonisation does not only constrain the smooth execution of adaptation initiatives, it also leads to fragmented and disjointed actions that limit the overall impact of these programmes [6]. Furthermore, the absence of robust monitoring and evaluation systems creates a substantial barrier [6, 72], impeding the ability to comprehensively assess the efficacy of implemented strategies and make informed decisions based on reliable data. This deficit in evaluation mechanisms undermines the adaptive capacity of health systems and constrains their ability to respond effectively to emerging climate risks. Moreover, limitations in organisational capacities within healthcare structures pose additional challenges to the successful implementation of health system adaptation strategies [54, 61, 73]. The inadequate infrastructure and organisational frameworks within these systems contribute to inefficiencies, hindering the prompt deployment of adaptive measures which impedes their effectiveness. Additionally, insufficient collaborative efforts across regions exacerbate these constraints [64], limiting the exchange of critical knowledge, resources, and sharing of best practices needed to bolster adaptation efforts. This lack of cohesive regional collaboration restricts collective ability to address climate-induced health risks comprehensively and compromises the overall resilience efforts of the health systems in the face of evolving challenges associated with climate change.

#### Low-risk perception and lack of expertise and evidence

Low-risk perception and lack of expertise are also barriers to health system response to climate adaptation. Low climate risk perception [15, 26, 35, 73], lack of knowledge and awareness about health risks from climate change [9, 26, 74], wrong perceptions about the health impacts of climate change [15, 54, 58], and a lack of awareness about heat stress among healthcare workers [59]affect perceived urgency for climate actions. Perceived lack of urgency [15], as well as expertise [39, 64, 71], and education make health system adaptation challenging, especially in developing countries [30]. Insufficient research [51], training [9], and access to climate data and models further hinder effective climate change response efforts of health systems [48]. Limited institutional capacity [48], gaps in reporting [6] and information [33, 68], and a lack of guidelines for reporting the health impacts of climate change [69, 72] present further challenges for health system adaptation.

#### Inequity and problems in healthcare delivery

Social inequality [58], maldistribution of adaptive capacity [54], and socio-political inequality [68] exacerbate existing health disparities. Besides, fragmented services for migrants [64], and socio-economic challenges [54, 73] contribute to further inequities which compromise the health systems’ response to the changing climate. Marginalised and hard-to-reach populations, especially in global south receive little consideration in climate change adaptation efforts [62, 72]. Moreover, lack of treatment protocols for illnesses related to extreme events [59] presents challenges for health system delivery. Shortages of staff [6, 59], difficulties in integrating evidence-based practices into healthcare [6], and a strained health system [62], especially in developing countries, impede effective health system adaptation to climate change.

## Discussion

We found that efforts are being made to build resilient health systems to climate risk by mainstreaming climate change in health policies and education, especially in developed countries. Investments are also being made in building climate-resilient health infrastructure, new technologies, robust early warning systems and surveillance programmes and quality health and climate research to inform health system climate actions. Attention has been given to improving the health and safety of health workers to improve quality healthcare delivery and access, especially for at high-risk communities and populations, including migrants. Also, investments are being made to train and improve the capacity of climate experts and institutions to produce high-quality evidence and national data systems that support health system adaptation decisions. However, inadequate funding, low climate risk perception, inadequate policy and poor policy implementation and evaluation, socio-economic challenges, lack of political support, deficits in evidence, and compromised healthcare systems, including infrastructure, make health system adaptation to climate risk challenging. Also, climate actions are not yet well integrated into mental health programmes, especially in vulnerable communities and populations.

### Health system response to climate change

The evidence produced in this review has significant implications for health systems and their adaptation to climate risk. Health system efforts to build resilience by mainstreaming climate change in health policies, planning and education, especially graduate and medical education, indicate an urgent recognition to address climate-related health impact. The other way is equally important, that health and health systems adaptation measures are incorporated into climate policies in dealing with the climate crisis. Thus, by integrating climate considerations into policy frameworks, health systems can ensure that anthropogenic climate change and its related health impacts are incorporated into planning and decision-making at all levels of governance and policy making [1, 2]. Furthermore, building robust health system infrastructures and surveillance systems are essential aspects of building resilience to climate risk. For instance, robust health infrastructures and other facilities can withstand the dangers pose by extreme weather events, ensure continuity of healthcare delivery, and avoid interruptions in health service delivery, especially for at-risk populations [18, 35, 70]. Early warning systems and new technologies, including remote sensing and enhanced testing and diagnostic facilities enable health systems to respond to climate risk promptly, to allow for early detection, prevention and management of climate-related diseases [33]. For instance, ensuring early detection of climate risk may allow for robust vector control and immunisation programmes [58, 74].

Climate change poses a significant risk to health workers, especially in outreach programmes in remote and hard-to-reach areas. Therefore, attention to improving the OHS of health workers is essential in improving access to quality healthcare delivery and helps eliminate all forms of precarious work conditions posed by climate change [4]. Health systems should thus, prioritise the well-being and safety of their workforce, as they are at the forefront of managing climate-related health emergencies. Thus, improving the quality of healthcare delivery and access ensures that all individuals, especially high-risk populations, including displaced and migrants, have equitable access to quality, culturally sensitive and patient-oriented healthcare services [61]. Such healthcare services can potentially address patients’ unique needs in the context of climate change [50]. Then, training and capacity building for healthcare workers, other experts and institutions have far-reaching implications. For instance, by improving institutional capacity to generate high-quality evidence and establish national data systems, health systems can make informed decisions, long-term plans and develop robust interventions that address climate risks [33]. Perhaps, evidence-based planning may help develop and implement contextually relevant adaptation strategies that are effective in protecting public health against climate risks [33, 47].

The co-benefits of climate adaptation through collaborative efforts of the health sector and non-health sectors are diverse [10]. While the primary focus is to address climate-related health risks, collective efforts can yield additional positive outcomes. For example, investing in climate-resilient infrastructures and promoting the use of renewable energy within the health systems can contribute to reducing GHG emissions, promote energy efficiency, and improve the overall sustainability of healthcare facilities and services [10]. Integration of climate risk into health policies and education fosters greater awareness and understanding among healthcare professionals, which enable them to advocate for sustainable healthcare practices and educate patients, families, and communities on climate-related health risks [10].

### Health system response in the realm of WHO’s Health System Resilience Framework

The imperative to build resilient health systems against the burgeoning impacts of climate change underscores the urgency to embed climate considerations within health policies, a notion that resonant with the WHO’s resilience framework. Integrating climate-related health impacts into decision-making at all governance tiers heralds a proactive stance in combating the multifaceted challenges posed by a changing climate [3]. Robust health infrastructures, bolstered by surveillance mechanisms, assumes a pivotal role in this framework, mirroring the WHO's tenet of resilient systems, capable of enduring extreme weather events and ensuring uninterrupted healthcare delivery, especially for vulnerable segments of the population [18, 35, 70]. Early warning systems and cutting-edge technologies, highlighted in this discourse, echo the WHO's focus on preparedness and swift response, essential for detection, prevention, and management of climate-driven ailments [33]. Furthermore, the emphasis on equitable access to quality healthcare, capacity building for healthcare professionals, and inter-sectoral collaborations to derive co-benefits mirrors the WHO's overarching aim of fostering sustainable healthcare practices, inclusive access, and bolstered readiness against the onslaught of climate risks [10, 33, 47, 50, 61].

The broader scope of our findings encompasses domains such as social support systems, One Health strategies, OHS and interventions related to sanitation, water, food, and mental health. The inclusion of One Health strategies underscores the interconnectedness of human, animal, and environmental health, recognising their interdependency in reducing climate-related health risks. Our study expands into the domain of social support systems. We highlight interventions targeting poverty alleviation, housing improvements for higher risk populations, and community-based support schemes. These interventions, albeit not explicitly outlined in the WHO framework, are vital for bolstering the adaptive capacity of health systems, especially in the face of climate-induced challenges. Furthermore, the attention to OHS in our research accentuates the significance of protecting healthcare workforce and adapt work processes to climate-induced stressors, to offer a comprehensive viewpoint beyond the WHO's workforce and governance components. In essence, our research extends the WHO framework by encompassing other critical dimensions and interventions crucial for health system resilience in the context of climate change. These expansions enrich our understanding and approach to addressing the multifaceted challenges posed by climate-related health risks, and pave the way for a more robust and inclusive response frameworks.

### Barriers to health system adaptation to climate risk

Inadequate funding, low climate risk perception, and poor policy implementation and evaluation are posing considerable challenges to health system adaptation to climate risk [73]. For instance, inadequate financial resources and political will limit the ability to invest in climate-resilient infrastructures, including new technologies and training programmes [27]. Moreover, low climate risk perception undermines the urgency and priority given to adaptation strategies in healthcare policy and planning [25, 63]. Unfortunately, inadequate policy and poor policy implementation and evaluation compromise the effective execution of climate-related health interventions while diminishing their impacts and potential co-benefits [10]. Meanwhile, socio-economic challenges including poverty and economic inequalities impede health system adaptation and push vulnerable people into extreme vulnerabilities. Displaced populations and migrants face disproportionate risks because they are less equipped to cope with climate-related health risks [8, 64]. Perhaps, inadequate funding and low climate risk perception hinder the integration of climate actions into OHS programmes, which compromise health workers’ well-being, safety and ability to respond effectively to climate-related emergencies [4]. Besides, lack of integration of climate actions into mental health programmes and services further suggests neglect of the potential psychological impacts of climate change. This situation reduces the resilience of high risk communities and populations facing climate crisis [75].

### Barriers in the realm of WHO’s Health System Resilience Framework

The identified barriers of health system adaptation to climate risk are intricately tied to the WHO 2015 Health System Resilience Framework, which reflects vulnerabilities across its core domains. Inadequate funding and limited financial resources align with the framework's pillar of Sustainable Financing. Insufficient funds impede investments in critical areas such as climate-resilient infrastructure, technology, and training programmes, directly affecting a health system's ability to withstand and respond to climate-related challenges [27]. Low climate risk perception resonates with the preparedness and emergency response domain of the framework. A diminished understanding of climate-related risks undermines the urgency and priority given to adaptation strategies in healthcare policies and planning [25, 63]. This lack of awareness hampers proactive measures, that hinder the system's readiness to effectively respond to climate-induced emergencies [3]. Poor policy implementation and evaluation directly impact the Governance and Leadership dimension of health system resilience. Moreover, ineffective policy execution limits the implementation of climate-related health interventions, reducing their potential benefits and co-benefits [10]. This inadequacy in policy implementation and evaluation compromises health system's ability to adapt and respond efficiently to the changing climate, also highlighting governance gaps and leadership deficiencies.

Moreover, socio-economic challenges, including poverty and economic inequalities, are closely related with the Social Protection and Equity element of the framework. These challenges exacerbate vulnerabilities, particularly among displaced populations, communities, and migrants [8, 64]. Such marginalised groups face heightened risks from climate-related health issues, because they may lack adequate resources and support structures proper resilience and access to quality healthcare [3]. Furthermore, the lack of integration of climate actions into OHS and mental health programmes underscores the need to bolster health workforce and service delivery within the resilience framework. Neglecting these aspects compromises health workers' well-being, safety, and ability to manage climate-related emergencies [4, 75]. Additionally, the oversight in addressing the psychological impacts of climate change reduces the resilience of high-risk populations, which highlights a critical gap in service delivery [75]. These barriers collectively underscore the intricate interplay between various dimensions of health system resilience, emphasising the necessity of comprehensive approaches outlined in the WHO framework to create a robust health system against climate risks and crises.

### Linkage between barriers and health system response to climate change

Understanding the intricate relationship between health system responses to climate change and the myriad challenges impeding such responses globally is imperative in comprehending the complexities faced in this arena. Examining the proactive measures adopted by health systems reveals a multifaceted approach which encompassess policy formulation, infrastructure enhancement, and community-focused initiatives [1, 10, 15]. These response strategies are in alignment with the guidelines provided by the WHO which aimed at fortifying and building robust health systems [3, 4]. However, impediments of substantial magnitude hinder these efforts significantly. A notable hurdle resides in the dearth of supportive policies, that render the implementation of response plans arduous [19, 71]. This critical gap also translates into a lack of emphasis on mental health concerns within these strategic frameworks [19, 59, 72].

While developed nations tend to prioritise disease-centric approaches and technological advancements [20, 39], they currently grapple with policy inadequacies, limited public awareness, and constraints in resource availability [15, 19, 27, 35, 71]. Conversely, developing nations place greater emphasis on community-driven strategies, educational initiatives, and cross-sectoral collaborations [33, 45]. Despite the divergence in their approaches, both categories of nations encounter similar challenges, notably the scarcity of financial resources and limited access to other vital resources [6, 15, 33, 45, 54, 71]. These challenges underscore the urgent need for sustained financial support and unwavering commitment from governments globally [9, 15, 26, 33, 35, 54, 61, 62, 71, 73].

Synthesising these diverse responses with the challenges at hand necessitates the formulation of comprehensive strategies that address these pressing issues [15, 19, 27, 71]. This comprehensive approach involves not only refining policies and enhancing financial resources, but also bridging the knowledge gap and fostering public awareness [15, 19, 27, 33, 71]. A holistic strategy that integrates these response initiatives while effectively navigating the barriers is pivotal in building resilient health systems to withstand the escalating impacts of climate change [15, 19, 27, 71].

### Recommendations for building resilient health systems

**Fig. 5:**
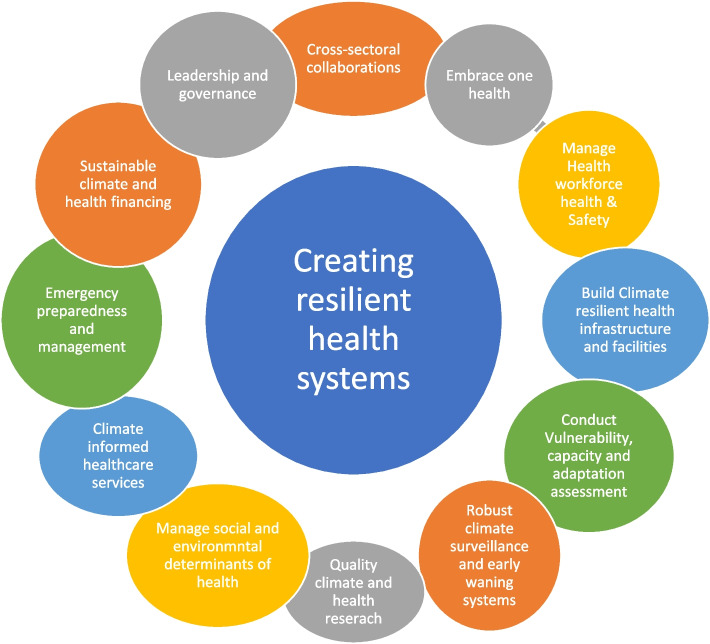
Recommendations for building resilient health systems

Recommendations for creating resilient health systems have been summarised in Fig. 5. These recommendations significantly bolster health system resilience, which align with the WHO’s Health System Resilience Framework of 2015. Such recommendations foster the capacity of health systems to prepare for, respond to, and recover from the adverse events of climate change [3]. Adequate and sustained financial backing is fundamental for health system resilience [3, 68]. For instance, allocating funds for climate-focused healthcare initiatives, infrastructure, research, and workforce training is pivotal. Such financing supports the development and maintenance of climate-resilient health systems, to ensure continuity in healthcare provision during climate-induced crises [2, 3].

Integrating climate considerations into healthcare services ensures responsiveness to climate-related health challenges. This involves creating services that are equipped to address climate-sensitive diseases, incorporate climate risk assessments into healthcare plans, and tailor health interventions according to changing climate patterns [3, 6, 15, 27, 39, 45, 68]. Robust emergency plans, that encompasses early warning systems, rapid response strategies, and community engagement, are vital components of resilient health systems [3]. Preparedness entails developing protocols for handling health crises linked to extreme weather events, infectious disease outbreaks, and other climate-related emergencies [3, 15, 45, 51, 68].

Effective governance structures and leadership are essential to foster resilience health system [3, 15, 24, 27]. This includes clear policy guidelines, coordination mechanisms across sectors, and the integration of climate and health priorities into national health agendas [3, 45]. Interdisciplinary collaborations across sectors are crucial. Engaging various sectors such as environment, agriculture, and urban planning facilitates a holistic approach to health system resilience [3, 35, 39]. This ensures shared responsibility and collective action in addressing climate-related health challenges.

This strategy ensures the comprehensive management of health risks arising from climate change, laying emphasis on the interdependence of health in different sectors. Ensuring the well-being of the health workforce involves addressing climate-induced health risks they may face [3, 15, 39, 70]. Providing adequate training, protective measures against climate-related hazards, and fostering a conducive work environment are pivotal. Moreover, infrastructure that can withstand climate-related stressors is vital [3, 27, 33]. Designing and maintaining climate-resilient healthcare facilities, equipped with adequate resources and utilities, ensures the uninterrupted delivery of health services during climatic disruptions. These recommendations collectively reinforce health system resilience that aligns with the WHO framework. It further focuses on the interconnectedness of health system components and emphasis preparedness, response, and recovery in the face of climate change challenges.

Developed countries should prioritise sustained investment in climate-informed healthcare services. This involves integrating climate change events into healthcare policies, to foster climate-resilient infrastructure, and conduct vulnerability assessments that address potential health risks from changing climate patterns [2]. Robust climate surveillance systems, akin to those recommended by the WHO [3], are essential to track and manage the health impacts of climate change. Early warning systems, informed by evidence-based research [3, 15], aid in proactive response measures against climate-related health hazards. Meanwhile, collaborative efforts, with various sectors, including environmental and urban planning agencies, are critical to ensure a comprehensive approach to a resilient health system [5].

In developing nations, enhancing health system resilience to climate change involves addressing resource scarcity and capacity gaps [6]. This includes increased funding for climate-resilient healthcare infrastructure [7], emphasising community-oriented healthcare services [8], and implementing adaptation strategies that consider local contexts and traditional healthcare systems [9]. Collaborative approaches to building resilient health systems, as advocated by the WHO [2, 3] require investments in workforce training and technology adoption [2, 27, 33]. Moreover, bolstering healthcare access for marginalised populations, tackling poverty and investing in social support systems are imperative in ensuring equitable health outcomes amidst climate challenges in developing countries [2].

### Limitations in the current review and recommendations for future studies

This review used only peer-reviewed journal papers published in English, which might affect the volume and depth of evidence retrieved. Excluding grey literature may affect the comprehensiveness and depth of evidence found in this review. Including grey literature such theses, policy documents, and other form reports could have given us a comprehensive data and findings. While we acknowledge this limitation, we attempted to minimise it by carrying out extensive consultations with key stakeholders in public health and health promotion. In addition, we thoroughly screened the reference lists of the full-text selected articles for other relevant records. Moreover, biases and other limitations inherent in the included articles may be carried in this review. However, the authors retrieved papers from 85 countries, including small island nations. We also failed to cover how health systems strengthening is included in the climate literature and climate policies. There is the need to explore how health systems strengthening is being integrated into climate policies. Furthermore, future studies should concentrate on using mixed-methods and grey literature to understand health system response and barriers, especially in developing countries where evidence remains scarce. Future research initiatives should prioritise investigating the responses of health systems to climate-related shocks, particularly focusing on areas such as 'One Health,' inequalities, and mental health. These facets have been relatively underexplored in current research and warrant attention due to their potential impact on healthcare resilience in the face of climate change.

## Conclusion

Efforts toward building resilient health systems, especially in developed nations, have seen promising strides by integrating climate change into health policies. Substantial investments in innovative technologies, early warning systems, and climate-resilient infrastructure reflect proactive measures in response to the climate risks. Concurrently, there is a concerted focus on enhancing healthcare delivery and access for high-risk populations, prioritising the well-being of healthcare workers, and fostering institutional capacity that generate essential evidence and robust data systems to navigate the challenges posed by climate change. However, significant obstacles persist in the adaptation of health systems to the climate risks. Challenges include a low perception of climate risks, inadequate policy implementation and evaluation mechanisms, and socioeconomic disparities. Particularly concerning is the limited integration of climate change into OHS programmes and mental health actions. These obstacles are predominant in developing countries due to resource constraints and weak healthcare infrastructures, which exacerbate vulnerabilities, and impede effective response to climate-related health risks. Addressing these multifaceted challenges is pivotal to create a resilient health system against the impacts of climate change and ensure the resilience of healthcare workers and at-risk populations. These pursuits resonate strongly with the WHO's health system resilience framework, which emphasis the importance of integrating climate change events into health policies and planning, strengthening healthcare delivery, fostering community engagement, and enhancing leadership and governance to navigate uncertainties and build resilient health systems against climate change challenges. Integrating climate change into OHS programmes, prioritising mental health actions, and bridging socioeconomic gaps are crucial facets that align with the framework's principles, which ultimately fortify health systems to withstand the complexities posed by the climate crises.

### Supplementary Information

Supplementary Material 1.

## Data Availability

All data generated or analysed during this study are included in this article and its Supplementary file (Table S1). Additional File (PRISMA_2020_Checklist) is also available.
