# Supplementary material for: Health systems response to climate change adaptation: a scoping review of global evidence
Source: BMC Public Health. 2024 Jul 29;24:2015. doi: 10.1186/s12889-024-19459-w (PMC11285469; doi:10.1186/s12889-024-19459-w)
Supplement: Supplementary file 1 — Supplementary Material 1. [file 12889_2024_19459_MOESM1_ESM.docx]

**Table S 1: Data extracted from reviewed studies**

| **Authors/ Country** | **Study purpose** | **Design** | **Health system response to climate change** | **Barriers to health system Adaptation** | **Quality appraisal** |
| --- | --- | --- | --- | --- | --- |
| [48]  USA | To discuss the role of public health in reducing human vulnerability to climate change. | Review | 1. By promoting safety and health to reduce the pre-existing burden of diseases, build social capital and strengthen community resilience to health hazards including extreme weather events. 2. Promotion of healthy people, healthy homes and healthy communities. 3. Preparing for extreme weather-related public health emergencies through risk assessment and emergency plans for long term sustained emergency operations. A public health related campaigns, public education and a well-established capacity for epidemiologic investigations. 4. Expected drought-related interventions relate to food insecurity, safe water and adequate sanitation, hygiene, infection control, surveillance and temporary shelter. 5. For wildfire, rapid assessment to identify gaps among health, medical needs and available resources; plays a role in healthcare delivery, assessment of sanitation and hygiene, food, water and air safety. 6. For flood related events, early warning systems, surveillance, basic rule of hygiene and sanitation are needed. Community primary care outreach are needed to overcome the barriers to access. Interventions are needed to evacuate special population such as those on admissions at hospitals, prisons, nursing homes, migrants, homeless and people with disabilities. | 1. Lack of supportive policy environment at national levels. | Moderate |
| [51]  USA | To determine the extent to which individual level attitudes and beliefs influence the adoption of climate change impacts. | Cross-sectional survey | 1. Educating the public about climate change on human health and safety. | 1. Low climate-related health risk perception, environmental attitudes and low efficacy. 2. Lack of political and financial support | Moderate |
| [65]  Brazil | To review data on how health care system and infectious disease control programmes can adapt to climate change. | Review | 1. Enhancement of epidemiological surveillance actions. 2. Development of early warning systems for epidemics, especially after extreme weather events. 3. Strategies to facilitate access to healthcare would assist early detection and treatment of infections. This should be directed to vulnerable populations and hard-to-reach areas. 4. Adaptation focused on specific disease and vector programs including entomological surveillance. | 1. Social inequality. 2. Wrong perception by decision makers that health risks caused by climate change are problem for the future and should have low priority in health policy. 3. Complexities of diseases transmission. | Moderate |
| [52] | To understand the role of health professionals in climate adaptation. | Review | 1. Public education 2. Preventive programmes e.g., vaccines, mosquito control, food hygiene and inspection and nutritional supplementation. 3. Healthcare for communities affected by environmental adversity. 4. Surveillance of disease (infectious disease). 5. Forecasting future health risk from projected climate change. 6. Forecasting future health risk and gains from mitigation and adaptation strategies. 7. Health sector workforce trained. 8. Early warning system for extreme weather events. 9. Neighborhood support schemes to protect the most vulnerable people. 10. Disaster preparedness. |  | High |
| [53]  Canada | To examine mainstreaming climate considerations into public health programs. | Mixed-method | 1. Risk management activities such as population health assessment, surveillance and public education and outreach. | 1. Unavailability of information about the linkage between climate change and health. | Moderate |
| [71]  USA | Assessment of local health department preparation for climate change-related health threats. | Qualitative |  | 1. Adaptation capacity decreased in several areas due to perceived lack of departmental expertise in climate change risk assessment. 2. Lack of departmental prioritization of adaptation; and the number of adaptation-related programs and services departments provided. 3. Funding constraints. | High |
| [8] | To analyse adaptative climate migration through public health lens. | Review | 1. Adopting a one health lens. 2. Facilitating organised relocation, including legal migration. 3. Offer climate-vulnerable individuals alternative employment-seeking channels. 4. To create a regulatory framework that responds to the implications of climate change for population health and health systems. To strengthen weak health systems in climate hotspots through capacity building and equality is the distribution of burden of care across healthcare system worldwide. 5. Strengthen human resource, infrastructure, and standards within healthcare systems are commensurate with population health needs. 6. Fostering preparedness for climate extremes. 7. Providing migrants with adequate nutrition, shelter, water, sanitation, hygiene, to reduce migrants’ vulnerability to infections. 8. Secondary screening of border crossers through well-regulated, legal migration procedures would still enhance early detection of illness, enabling appropriate early referrals for necessary health services. 9. Working to provide legal status to climate migrants to be given legal status through appropriate coordinated immigration procedures to enable them access healthcare services. 10. Health awareness 11. Equitable resource and power distribution. |  | High |
| [37]  Australia | To examine the linkage between climate change and human health and building Australia’s capacity for adaptation. | Review | 1. Prepare health services for predicted health risks from heatwaves, bushfires, infectious diseases, diminished air quality, and the mental health impacts of climate change. 2. Health system should be flexible and robust, with resources strategically allocated. 3. Long term planning will also require close collaboration with non-health sector and adopt one health. |  | Moderate |
| [54]  Australia | To examine the increased attention and adaptation of healthcare facility infrastructure. | Review | 1. Increased public awareness. 2. Warning procedures for the community especially those at higher risk. 3. Coordinated disaster responses – emergency services – ambulance, fire, police, etc. that work with facilities to ensure healthcare delivery is uninterrupted. 4. Relief plan: surge hospitals, counselling, etc. |  | Moderate |
| [15]  USA | To examine public health climate change adaptation planning. | Cross-sectional surveys | 1. Monitoring, Surveillance, and Community screening. 2. Coordination and collaboration 3. Education and awareness creation 4. Policies and planning. 5. Research and innovation 6. Workforce development. 7. Access to health service. 8. Program evaluation. | 1. Lack of incorporating climate and health into planning. | High |
| [32] | To examine health sector strategies for mitigation and adaptation. | Review | **Mitigation**:   1. Building green which includes strategies to conserve energy. 2. Efficient energy distribution and use of renewable energy sources. 3. Passive or low energy cooling, heating and ventilation strategies. 4. Strategies for conserving and maintaining water resources. 5. Reducing GHG emissions from anesthetic gas use and waste management. 6. Increased use of health information technology, such as eHealth.   **Adaptation:**   1. Telemedicine in disasters. 2. Point-of-care diagnostics for diseases outbreaks. 3. Public health surveillance of disease and mapping strategies using mobile devices. 4. Remote education in preparedness and adaptation for health professionals and the public. | 1. Lack of infrastructure for robust mobile communications. 2. Slow or lack of improvement in disaster preparedness in hospitals and out-patient clinics. 3. Lack of alternative and reliable energy supplies e.g. solar panels. 4. New technology remains under development, costly and potentially unreliable. 5. Lack of skilled workforce. 6. Lack of education for increased knowledge and preparedness. | High |
| [55]  Japan | To examine public health adaptation strategies to climate related heat stroke. | Review | 1. Alert system through automated WBGT monitoring system at elementary schools and other early warning systems. 2. Health related health information and education plan. 3. Reduction in indoor heat exposure through cultivation of climbing plants on facades. 4. Care for vulnerable people. 5. Preparedness of the health and social care system. 6. Surveillance, monitoring and evaluation. | 1. Scarcity of locally-relevant evidence. 2. Lack of expertise | Moderate |
| [27]  Germany | To explore the concerns, needs, and expectations of inpatients with the goal to develop a patient-centered climate change adaptation agenda for hospitals | Qualitative | 1. Focusing on patient-centered care including establishing “caring” medical services and a health promoting environment. 2. Improve ventilation, air conditioning and ensuring thermal comfort. 3. Infrastructural improvements and integrating the hospital into the urban environment. 4. Intensified education which includes education of staff and patients in climate change. 5. Improvement of staff number and work place ergonomics. 6. Using the potential of climate change adapted work processes and individual patient behaviour. |  | Moderate |
| [56] | Identifying practical adaption options to climate change for health sector | Review | 1. Emergency preparedness. 2. Implement early warning systems and emergency response plans. 3. Conduct education and outreach on emergency preparedness and exposure to air pollutants as well as heat waves. 4. Vaccinate public for diseases they are likely to be exposed to. 5. Provide low-cost vaccinations to those likely to be exposed. 6. Develop robust diagnosis for food and waterborne diseases. 7. Sponsor research and development on vaccines and other preventive measures. |  | Moderate |
| [21]  India | To explore healthcare system preparedness and adaptation planning for changing climate. | Mixed-method | 1. Making public health infrastructure more resilient to the impact of heat stress. 2. Integrating health adaptation into national health planning. 3. Maximizing synergies across sectors. | 1. Limited plans and programmes to tackle the health risks of extreme weather events. 2. Treatment protocols do not exist for all illnesses and injuries that tend to occur during extreme events. 3. Minimal awareness about heat stress among healthcare providers and remote communities. 4. No inclusion of resources planning in disaster response. 5. Shortage of staff 6. Poor infrastructure. 7. Lack of surveillance on vulnerabilities. | Moderate |
| [20]  Botswana,  Egypt,  Eritrea,  Gambia,  Ghana,  Guinea-Bissau,  Kenya,  Lesotho,  Malawi,  Mauritius,  Namibia,  Nigeria,  Sierra Leone,  South Africa,  Sudan,  Swaziland,  Seychelles,  Uganda,  Zambia,  Zimbabwe | To examine climate change adaptation measures in African public health sector | Review | 1. Develop early warning system. 2. Infectious disease control and improving vaccination programmes. 3. Public education and awareness. 4. Promoting appropriate climate-health education in schools. 5. Improving waste collection and environmental sanitation. 6. Surveillance, research and monitoring. 7. Mainstreaming climate change in health policies and strategies. 8. Improving public health infrastructure and technology. 9. Improving housing systems for lower-income families. 10. Investing in clean water technologies. |  | High |
| [29]  Australia  Belgium  Canada  France  Ireland  Luxembourg  New Zealand  Switzerland  UK  USA | To explore public health adaptation to climate change in OECD countries | Review | 1. Inter-sectoral adaptation planning. 2. Educate health professionals about the health impact of climate change (FR, BE). 3. Heat risk adaptation guidelines for public health and emergency management CA). 4. Raise awareness of climate change impacts and social vulnerability (IR). 5. Strengthening network of expertise and centres at national and international levels (SW). 6. Establishing internal multidisciplinary work of group to investigate the occupational safety and health implications of climate change (US). 7. Heat wave plan (UK) 8. Eradication of Aedes japonicus mosquito (BE). 9. Stockpile critical medical supplies and pharmaceuticals (US). 10. Identify the capacity of the public health system and hospital system to plan and respond to vulnerabilities (AU). 11. Research the potential effect of weather patterns and climate on outbreaks of environmentally sensitive infectious diseases (US). 12. Surveillance for heat response plan (LU). 13. Food and water-borne infectious diseases surveillance (NZ). 14. Maintain and expand real time UV monitoring. | 1. Almost half of the health adaptation initiatives planned or implemented do not target specific health risks 2. Mental health and associated adaptation policy action received minimal attention. | High |
| [38] | To examine present and future risks to health and necessary response to | Review | 1. Well-coordinated intersectoral action. 2. Widening the geographical range of infectious disease surveillance programmes. 3. Improving infrastructure and capacity of institution such as hospitals, ambulance services, etc. to respond to the impact of extreme weather events. 4. Creating awareness and educating communities and families. 5. Reinforce society’s commitment to undertake climate mitigation actions by promoting positive message that climate actions will yield. |  | Moderate |
| [49]  Poland | To examine healthcare system adaptation to heat stress. | Mixed-method | 1. Fitting air-conditions in hospitals 2. Capacity building for staff for extreme weather events including heat stress. 3. Storage of seasonal supplies in sufficient quantity. 4. Adequate protection of workplaces exposed to climate factors. 5. A warning system for society and healthcare authorities of extreme weather events. 6. Periodic courses to upskill medical personnel in diagnosis and treatment of heat-related disorders. 7. Public education, and promotion of healthy nutrition, lifestyle and appropriate reaction to extreme weather events. 8. Modernisation of laboratories for early diagnosis. 9. Improvement of healthcare infrastructure. 10. Surveillance 11. Funding for research on epidemiology, toxicology and climate physiology. |  | High |
| [6]  Fiji  Kiribati  Marshall Islands  Micronesia  Nauru  Palau  Papua New Guinea  Samoa  Solomon Islands  Tonga  Tuvalu  Vanuatu | To examine health system intervention strategies to climate response. | Review | 1. Training the workforce on managing climate related health risk. 2. Promoting climate informed health programmes. 3. Integrating climate risk monitoring in health programmes such as the early warning signs. 4. Cross-sector coordination. 5. Translating research into practice. 6. Impact evaluations and dissemination of studies | 1. Shortage of trained health staff. 2. Overload of information and reporting. 3. Ineffective coordination mechanisms. 4. Unsuccessful implementation of interventions. 5. Difficulty mobilizing resources. 6. Gaps in reporting and evaluation. 7. Taking longer years of incorporating evidence-based practices on climate change into general healthcare practice and specific health programmes. | High |
| [22]  Canada | To examine public health sector adaptation to climate change. | Qualitative | 1. Mainstreaming climate into existing public health programmes. 2. Local health system adaptations. 3. Surveillance. 4. Building awareness effectively in local and diverse communities. 5. Improvements in healthcare infrastructures to be resilient. 6. Assessments of health impact. 7. Facilitate community climate change knowledge sharing and planning. 8. Improving local capacity. 9. Evaluating for effectiveness of climate adaptation programmes. | 1. Lack of resources and long-term funding. 2. Lack of urgency. 3. Lack of data on causal estimates of morbidity and mortality associated with weather events. 4. Climate change has not resonated in communities. 5. In Effective messaging is a challenge in diverse communities. 6. The public lacks a clear perception of personal risk | High |
| [61]  Tanzania | To examine the impact of climate change on health and health system. | Review | 1. Emergency preparedness and response to address all health aspects of emergency and disaster. | 1. There is no single policy document that is solely responsible for climate change in Tanzania. 2. Little research and strategic implementation. | Moderate |
| [39]  SSA | Examine health system adaptation climate related migration | Review | 1. Strengthening health system. 2. Providing access to healthcare. 3. Culturally-appropriate services. 4. Policy oriented research and training. 5. Inter-sectoral collaboration. 6. Provision of emergency services 7. Public health services such as immunization and mother, and child preventive services. | 1. Fragmented services for migrants. 2. Acute shortage of resources. 3. Not prepared for the burden of climate migration 4. Lack of investment in health system. | Moderate |
| [30]  Cape Verde  Comoros  Mauritius  Sao Tome and Principe  Seychelles  Bahrain  Cook Island  Fiji  Kiribati  Maldives  Marshall Islands  Micronesia  Niue  Palau  Papua New Guinea  Singapore  Solomon Islands  Tonga  Tuvalu  Vanuatu  Cyprus  Malta  Antigua and Barbuda  Bahamas  Barbados  Cuba  Dominica  Dominican Republic  Grenada  Haiti  Jamaica  Saint Kitts and Nevis  Saint Lucia  Saint Vincent and the Grenadines  Trinidad and Tobago | To explore existing interventions to reduce climate-sensitive diseases. | Review | 1. Development of early warning system. 2. Surveillance. 3. Evaluation of surveillance and warning systems. 4. Building institutional, human and scientific capacity for flexible and responsive action. 5. Vulnerability assessment. 6. Health education campaigns. 7. Improvement of health and sanitation infrastructure. 8. Promoting research. 9. Strengthening vaccination programmes. 10. Improving water systems. 11. Designing of health infrastructure to reduce climate impact. 12. Sea defense projects 13. Enforcing climate friendly land use policies and regulations. |  | High |
| [31]  USA | To examine public health adaptation to climate change. | Review | 1. Improve health system preparedness to extreme weather events. 2. Implementing new and effective warning systems. 3. Ensure health supply chains are improved. 4. Strengthen infrastructure resilience to sea level rise. 5. Continuously update and improve emergency risk communication with focus on vulnerable people. 6. Education and capacity building for communities and staff. 7. Facilitate access to community education programmes about heat risk and promote better understanding of heat warnings and awareness. 8. Develop air conditioning programmes for low-income households and individuals at risk for heat-related illness. 9. Implement Surveillance systems for climate sensitive diseases. 10. Enhance health system capacity for rapid disease-specific emergency response. 11. Timely relocation of displaced individuals and migrants. 12. Maintenance of social structure for forced migration. |  | High |
| [68]  USA | Examine how mental health can be integrated into climate change and health vulnerability assessment. | Review | 1. Mapping and monitoring psychosocial resources and skills within affected communities. 2. Mapping inter-disciplinary relationships that can enhance psychosocial response. 3. Providing skills, resources and critical information to support mental health adaptation. |  | Moderate |
| [57]  Canada | Explore health system adaptation to climate change. | Review | 1. Enhance public health surveillance, monitoring and early warning systems. 2. Emergency preparedness and education. 3. Promote traditional health systems. 4. Poverty alleviation programmes. 5. Access to clean water, food and safe shelter. 6. Provide culturally appropriate health services. 7. Promote traditional knowledge 8. Enhance local and regional health practitioners’ capacity about climate change and related impact. |  | Moderate |
| [64] | To outline changes in public health practice that could increase public health’s adaptive capacity. | Review | 1. Impact assessment. 2. Vulnerability assessment and mapping. 3. Hazard mapping. 4. Decision support tools. 5. Integrated assessment model. 6. Early warning systems. 7. Syndromic surveillance. 8. Remote sensing. 9. Problem-based learning. | 1. Uncertainty in climate projections. 2. Socio-economic challenges. 3. Financial challenges 4. Maldistribution of adaptive capacity. 5. Limitations in technological advancement. 6. Institutional arrangements that limit collaborative efforts and accumulation of evidence about effective adaptation. 7. Limit on social capital on community level adaptation. 8. Inaccurate perception or uninformed perception of individuals. | Moderate |
| [9]  Dominica republic | Explore health adaptation challenges. | Mixed method | 1. Public health surveillance. 2. Water quality monitoring. 3. Food handler’s certification programmes. 4. Community clean-up campaigns. 5. Solid waste collection and management. 6. Health messaging during critical periods. 7. Health promotion through radio and television channels. 8. Mosquito control programmes. 9. Early warning systems. 10. Smart hospital initiative. 11. Disaster emergency management. 12. Medical information system project. 13. Enhance enforcement of existing legislation. 14. Provide training to health sector staff on health impact of climate change. 15. Enhance the integration of climate services into health decision making. 16. Ensure sufficient resources for emergencies. 17. Provide funding and resources for climate research, food borne disease, water-borne disease, vector-borne disease and food security. 18. Develop national electronic database for climate change and related issues. 19. Ensure mechanism for routine flow of information and cross-sectoral collaboration. 20. Routine surveillance. 21. Increase climate resilience health facilities and infrastructure. 22. Increase capacity of laboratory facilities. | 1. Insufficient training of stakeholders about climate health risk and adaptation needs. 2. Insufficient knowledge of health risks from climate change. 3. Lack of financial resources. 4. Lack of human resources. 5. Uncertainty about the best adaptation options. 6. Lack of local data to inform development of adaptation options. | High |
| [40]  Indonesia | To explore public health response to climate change impact. | Review | 1. Health promotion. 2. Relocating population. 3. Surveillance. 4. Prevention and preparation for communicable disease control. 5. Health workforce training. 6. Intersectoral collaborations. |  | Moderate |
| [67]  Indonesia | Explore primary healthcare adaptation strategies | Review | 1. Performing eradication of mosquito nets at household level in community. 2. Delivering health promotion and targeted water and sanitation activities. 3. Visiting home care particularly for vulnerable groups in community such as pregnant women, breastfeeding mothers, children, elderly, disabled persons and chronically ill persons. |  | Moderate |
| [69]  USA | To explore ways to prepare health system to respond climate change. | Mixed-method | 1. Adding chillers to emergency power. 2. Creating street connections for supplemental emergency power and chillers. 3. Energy efficiency initiatives to reduce burden on power sources in current and future facilities. 4. Business continuity planning. 5. Flood barriers. 6. Utility hardening. 7. Storm water management system. 8. Advocate and partner for public utility improvement. 9. Incorporate flood projections into new building designs and relocations. 10. Roofing repair and replacements. 11. New facilities built to codes of areas with higher wind speeds. |  | High |
| [50]  UK | Explore the impact of extreme weather events for health and social care system. | Review | 1. Changes to built infrastructure systems such as transport, utilities and individual care facilities. 2. Institutional and social infrastructure supporting health care system. 3. Organisations, communities and individuals need to adapt their practices to improve resilience of health and health care to extreme weather. 4. Preparedness and emergency response strategies call for action extending beyond the emergency response services, to include health and social care providers more generally. |  | Moderate |
| [23]  Cooks Island  Federated States of Micronesia  Fiji  Kiribati  Marshall Islands  Nauru  Niue  Palau  Samoa  Solomon Islands  Tonga  Tuvalu  Vanuatu | To explore climate change adaptation priorities. | Review | 1. Ensuring that health and safety consideration are incorporated into adaptation activities across sectors. 2. Improving the safety and security of food and water. 3. Improving sanitation and hygiene facilities. 4. Increased resources for health emergency risk management. 5. Climate-proofing key health and safety infrastructure. 6. Enhanced surveillance targeting climate-sensitive disease and their risk sources. 7. New and improved communication pathways between the health sector, metrology services and other stakeholders. 8. Early warning systems. |  | High |
| [16]  Vietnam | To explore healthcare adaptation to climate change. | Mixed method | 1. Strengthen capacity of all staff at different levels through training and capacity building. 2. Improving awareness and capacity of the communities in health adaptation through risk communication activities. 3. Develop and implement early warning systems. 4. Promote intersectoral and international collaboration. 5. Strengthen scientific research. 6. Strengthen infrastructure and equipment capacity to increase health facility resilience to extreme events including heat waves. 7. Adapt health system to improve management of climate sensitive diseases. 8. Develop and refine regulations, policies and mechanisms of the health sector in managing the health risks of climate change and extreme weather events. |  | Moderate |
| [24]  Canada | Explore public adaptation to climate change in Canada | Review | 1. Develop human resources, institutions and communities, and equip them with the capacity to adapt to climate change. 2. Incorporating understanding of climate science, impacts and vulnerability and risk into government and health system planning, management, policies and regulations. 3. Explore local and evidence-based adaptation resilience such as ways to eradicate mosquitoes and increasing tree canopy. 4. Building digital database of climate change, its impact and related information. 5. Effectively communicate climate information to help build resilience. 6. Investing and implementing new and enhanced early warning systems for communicating weather and climate risk. 7. Surveillance and vulnerability assessment. |  | Moderate |
| [17]  Australia | To explore climate change impact and adaptation opportunities. | Review | 1. Developing policies that promote social capital and cohesion in cities. 2. Health promotion should reach those in need for behavioural change to reduce chronic disease burden. 3. Integrated approach to urban design and infrastructure. 4. Improve water sources. 5. Improve health infrastructure. 6. Invest in early warning systems. 7. Surveillance of health impact of climate change. 8. Enforcing regulations on proper food handing and storage alongside public education campaigns. |  | Moderate |
| [25] | To assess international adaptation to climate change. | Review | 1. Improving evidence and understanding of the current associations between climate and health outcomes including health and populations most vulnerable. 2. Providing health and emergency management officials, stakeholders, and the public with information on the magnitude and pattern of current and future health risks associated with climate variability and change. 3. Identifying vulnerabilities in the health system. 4. Incorporate climate change concerns into existing and new health policies and planning. 5. Forging collaborations with sectors such as water and sanitation infrastructure and management. 6. Strengthen the case for investment in health protection and promotion. | 1. Difficult access to climate data 2. Limited number of climate and health models. 3. Uncertainty in climate projections. 4. Lack of funding. 5. Lack of expertise in developing countries. | High |
| [46]  2014 | To examine weather evidence-based public health can be applied to climate change adaptation. | Review | 1. Increased attention to designing, evaluating and reporting adaptation intervention. 2. Standardized health impact projection reporting, 3. Increased attention to knowledge brokering or translation. | 1. Lack of higher level of evidence of intervention efficacy. 2. Lack of guidelines for reporting climate change health impact projections. | Moderate |
| [62] | To review health system adaptation to climate change. | Reviews | 1. Improved and increase surveillance for infectious disease particularly for diarrhea, malaria and dengue. 2. Develop community-based models of management of children with acute malnutrition 3. Plan emergency response. 4. Improved water and sanitation facilities. |  | Moderate |
| [10] | To examine the co-benefits of urban climate adaptation. | Review | 1. Maintaining and upgrade water treatment, sewage and sanitation facilities. 2. Upgrading health infrastructure. 3. Improving ventilation e.g. air conditioning. 4. Integrated approach across all sectors. 5. Prioritising equity in health adaptation planning. 6. Communication of co-benefits and actions to protect health. 7. Support social networks. 8. Early warning system. 9. Response plans to protect vulnerable groups. 10. Improve local monitoring of climate parameters. |  | High |
| [73] | To explore constraints and barriers to public health adaptation to climate change. | Review |  | 1. Uncertainties of future climate and socioeconomic conditions. 2. Financial challenges 3. Technologic limits. 4. Institutional arrangements. 5. Fragmentation and policy contradictions. 6. Paucity of knowledge about potential effectiveness of different adaptation options. 7. Individual cognition. | Moderate |
| [72] | To explore public health adaptation to climate change. | Review |  | 1. Limited evidence of reporting of institutional adaptation at the municipal level in urban areas in Global south. 2. Lack of information-based adaptation initiatives. 3. Limited focus on initiatives addressing infectious disease risk. 4. Absence of monitoring, reporting and evaluation. | Moderate |
| [35]  Peru | To understand the role and priority status of health within the adaptation planning and process. | Review | 1. Capacity building programmes for technical assistance to the regional health directorate and local establishments. 2. Improve health care infrastructure and facilities. 3. Strengthening of epidemiology and sanitary surveillance and health services. 4. Implementation of nutritional practices in the face of climate change. 5. Improve universal health care access. 6. Strengthen capacity of public health. 7. Adequate allocation of resources. 8. Promote both economic development and preventative health in rural areas. 9. Reduce health effect of the most vulnerable population. 10. Conduct research o vulnerabilities in the health sector. 11. Dissemination of knowledge, socialization on climate change impact on health. 12. Strengthen epidemiological vigilance and health services to confront emerging diseases. 13. Develop research on the coverage of the health sector and health in hard-to-reach areas. 14. Capacity building and knowledge dissemination for health personnel. 15. Strengthening community programmes and services. 16. Strengthen regional governance and public health policies. 17. Reduce the health effect of children under-five. 18. Expansion of emergency services to include children under-5. 19. Reduce poverty. 20. Combating zoonotic disease. 21. Technical capacity building of health professionals. 22. Improvement of communications and sensitization of preventive projects. 23. Improvement of access to pharmaceuticals for increased health risk. 24. Installing of early warning system. 25. Improve the capacity and quality of care of the health sector. | 1. The national adaptation plans do not include health as a priority. 2. No cohesiveness between plans in format, content, planning and execution. 3. Little consideration for marginalized populations. 4. Lack of local research on health impacts of climate change. | High |
| [36]  Brazil  Colombia | To explore healthcare adaptation plans to climate change. | Review | 1. Ensuring healthcare services are accessible to all including vulnerable population without discrimination. 2. States must provide health care services that are available in sufficient quantity; physically, geographically, and economically accessible; culturally appropriate; gender sensitive; medically, ethical; and of good quality. 3. States have an obligation to ensure access to climate-related health information, particularly among those living in vulnerable circumstances. 4. Monitoring and evaluation. |  | Moderate |
| [33]  Australia | To explore climate adaptation by local health directorate. | Review | 1. Monitor severe weather predictions to inform emergency response planning. 2. Compile data on the correlation between extreme events and demand to inform workforce and resource planning. 3. Knowledge dissemination to communities and households. 4. Incorporating health services into government adaptation planning. 5. Support health staff to collaborate on research which local universities on climate change adaptation. 6. Review cooling capacity in all health facilities. 7. Heat management plans for all staff, especially if working outdoor. 8. Incorporate climate change adaptation to occupational health and safety assessment. 9. Prepare, inform and debrief staff on extreme events. 10. Incorporate climate change adaptation planning into financial planning. 11. Review insurance policies to cover extreme weather events for health workers. 12. Explore alternative of-the-grid solutions |  | Moderate |
| [34]  Australia  New Zealand | To examine hospital adaptation to climate change | Qualitative | 1. Effective communication and coordinated response and feedback mechanism in responding to future events. 2. Maintain essential services. 3. Maintain physical integrity of the health infrastructure or facilities. 4. Access to transportation to increase access to healthcare. 5. Staff availability and safety to support continuity of service delivery. |  | Moderate |
| [70]  Canada | To explore vulnerabilities of Aboriginal health systems in Canada to climate change. | Review |  | 1. Economic poverty. 2. Technological constraints. 3. Socio-political value inequality. 4. Constrained institutional capacity. 5. Information deficit. | High |
| [74]  Israel  Jordan  West Bank  Gaza | To explore lessons from climate change adaptation | Review |  | 1. Limited strategy, capacity and resources for health system preparedness. 2. Unclear long-term planning. 3. Health inequalities. 4. Limited regional collaborations. 5. Lack of political will. 6. Low awareness about the impact of climate change. | High |
| [47] | Explore public health response | Review | 1. Monitor health status to identify and solve community health problems. 2. Diagnose and investigate health problems and health hazards in community. 3. Inform, educate and empower people about health issues. 4. Mobilise community partnership and action to identify and solve health problems. 5. Develop policies and plans that support individual and community health efforts. 6. Enforce laws and regulations that protect health and ensure safety. 7. Link people to needed personal health services and ensure the provision of healthcare. 8. Ensure consistent public and personal healthcare workforce. 9. Evaluate effectiveness, accessible and quality of personal and population-based health services. 10. Research for new insights and innovative solutions to health problems. |  | Moderate |
| [66]  South Africa | To explore the role of health sector in climate change adaptation | Review | 1. Early warning system. 2. Surveillance for malaria and infectious disease. | 1. Policies pay little attention to health concerns and the specific needs of vulnerable groups. 2. Little efforts on preparedness for extreme weather events. 3. Strained health system. 4. Few adaptation measures in occupational health and safety. 5. Political barriers. 6. Climate change is not in the mainstream curricula of medical school. | High |
| [41]  Ghana  Nigeria  South Africa  Sudan  Uganda | To examine primary healthcare adaptation | Review | 1. Training and retention of competent workforce 2. Community-oriented approach to healthcare 3. Provide early warning systems 4. Strengthen multi-sectoral collaboration 5. Educate public health workers 6. Develop resilient and adaptative public health infrastructure 7. Healthcare facilities should include renewable energy sources. 8. Back-up generator for emergency situations. |  | High |
| [26] | To explore health system adaptation and mitigation strategies | Review | 1. Health should be incorporated into all policy creation and implementation with public health scientists and health care professionals engaged at each stage of policy development. 2. Prioritize climate action within their cities, states, nations, and across borders focused on environmental justice and advancing the health of vulnerable populations. 3. Climate education should be incorporated into schools and graduate studies to ensure a basic foundation of science across sectors and critical thinking skills are built. 4. Provide adequate funding for quality research. 5. Collaboration across multiple sectors |  | High |
| [45]  Iran | To examine health system plan for implementing Paris agreement. | Qualitative | 1. Identifying vulnerable groups 2. Assessing vulnerability 3. Increasing capacity of health services during extreme events 4. Early warning system 5. Using new technologies to increase adaptation 6. Financial support 7. Increasing the number of researchers 8. Increasing knowledge and skill of staff 9. Public awareness. 10. Using renewable energy | 1. The health services contribute low to climate mitigation. 2. Lack of data | High |
| [18]  Ghana, Nigeria, South Africa, Namibia, Ethiopia, and Kenya | To evaluate the levels of information, knowledge, and perceptions of public health professionals | Mixed method | 1. Increasing progress in policy frameworks 2. Integrate climate change into medical school curricula 3. Progress in surveillance system 4. Training curricula for health workers. 5. Educating health personnel on climate change. 6. Incorporate climate change management when implementing public health interventions. | 1. Low skilled personnel | High |
| [58]  Philippines | To explore ethe role of health professionals in leadership | Review | 1. Obstetrician/gynecologists have the unique opportunity to raise awareness, educate, and advocate for mitigation strategies to reverse climate change affecting our patients and their families |  | Moderate |
| [19]  China | Exploring climate change adaptation actions | Review | 1. Increase adaptation across governmental departments and accelerate investment in climate resilience. 2. Develop a stand-alone heath national adaptation plan for climate change. 3. Prioritize climate change in health policies with focus on well-being of vulnerable population. 4. Increase awareness within the healthcare sector of the threats to health that climate change will bring. 5. Provide vulnerable population with early warning information services. 6. Provide guidelines for safe outdoor physical activity under heat stress. 7. Provide community-based health education for older population. 8. Establish a regional health and ageing dataset for intervention purpose. 9. Hospitals, Red Cross Society of China should collaborate. |  | High |
| [42]  USA | To explore progress of health departments towards adaptation. | Mixed method | 1. Leverage on strategic collaborations 2. Extensive public education and communication campaigns. 3. Development of political capital. |  | High |
| Banwell et al., 2018 | To examine climate change adaptation in health | Review | 1. Early warning system 2. Vulnerability and risk assessment. 3. Health system strengthening. 4. Infrastructure resilience. 5. Disaster preparedness and response. 6. Health impact pathways. 7. Build existing expertise and resources 8. Risk communication |  | High |
| [43]  Sweden,  Portugal  Philippines | Explore options and challenges to health adaptation to climate change ` | Qualitative | 1. Collaboration between health sector and non-health sectors 2. Water and sanitation improvement. 3. Prevention of heat waves in vulnerable groups | 1. Insufficient financial resources 2. Limited inter-sectoral collaborations 3. Lack of involvement of public health sector in adaptation process. | High |
| [28] | To explore challenges and opportunities for advancing work on climate change and public health | Qualitative | 1. Integrating climate change into current public health practices. 2. Providing inter-sectoral support for climate solutions with health co-benefits 3. To engage and mobilize communities through education and communication 4. Increase funding 5. Increase work on the shared root causes of climate change and inequities. | 1. Low risk perception 2. Insufficient understanding of climate change’s health impact. 3. Lack of public health capacity 4. A narrow framework for public health practice that limit works on the root causes of climate change and health. | Moderate |
| [44] | To examine how to integrate public health into climate change policy and planning. | Review | 1. Monitoring climate hazards 2. Diagnosing health status 3. Assessing vulnerability 4. Mobilizing partnerships 5. Communication of climate risk 6. Workforce development 7. Evaluation |  | High |
| [60]  Australia | To explore how to prepare the health services for climate change | Review | 1. Improve upon existing services 2. Prioritize vulnerable communities and populations. 3. Ensure equitable access to health services. 4. Improve infrastructure 5. Ensure back-up power supply. 6. Make available rapid testing and diagnosis in vulnerable communities and regions. 7. Sustain health workforce 8. Ensure consistent workforce 9. Prepare frontline health workforce o be prepared to manage heat stress. 10. Deliver education to individuals, families and communities. 11. Increase awareness of the mental health effect of climate change among health workers including psychiatric and Para psychiatric services. 12. Mental health workforce should educate communities most vulnerable to climate change. 13. Invest in areas where mental health services are under-resourced and where menta health effect will be substantial. |  | Moderate |
